# The Elusive Fraction of Marine Plankton Diversity: Size, Abundance and Taxonomic Composition of 0.2 μm‐Filterable Prokaryotes Across Three Contrasting Coastal Environments

**DOI:** 10.1111/mec.70434

**Published:** 2026-06-15

**Authors:** Clara Ruiz‐González, Cristina Andres‐Barrao, Anders Lanzén, Laura Alonso‐Sáez, Hugh Ducklow, Josep M. Gasol, Xosé Anxelu G. Morán

**Affiliations:** ^1^ Institut de Ciències del Mar (ICM‐CSIC) Barcelona Spain; ^2^ Biological and Environmental Science and Engineering (BESE) Division King Abdullah University of Science and Technology (KAUST) Thuwal Saudi Arabia; ^3^ KAUST Beacon Development (KBD) KAUST National Transformation Institute (NTI) Thuwal Saudi Arabia; ^4^ AZTI Marine Research, Member of Basque and Technology Alliance (BRTA) Sukarrieta Spain; ^5^ IKERBASQUE Basque Foundation for Science Bilbao Bizkaia Spain; ^6^ Lamont‐Doherty Earth Observatory Columbia University Palisades New York USA; ^7^ Centro Oceanográfico de Gijón/Xixón (IEO, CSIC) Gijón/Xixón Spain

**Keywords:** 0.1 μm filter, marine prokaryotic communities, size‐fractionation, starvation forms, ultramicrobacteria

## Abstract

The existence of prokaryotes escaping filtration through 0.2‐μm filters has been known for decades. Such ‘filterable’ prokaryotes (hereafter ‘FP’) might include true ultra‐small groups (i.e., ultramicrobacteria), but also cells that miniaturize temporally as a physiological strategy to persist under unfavourable conditions, representing a ‘seed bank’ that can flourish when conditions change. However, the taxonomy, environmental drivers and ecological relevance of these microorganisms remain unknown due to a scarcity of studies targeting this size‐fraction. We characterized prokaryotic assemblages from the 0.1–0.22 μm and the > 0.22‐μm (‘0.2RP’ for 0.22‐μm‐retained prokaryotes) size‐fractions across different sites in the NE Atlantic (Bay of Biscay), the NW Mediterranean Sea and the Red Sea. 9%–15% of total cells passed through 0.22‐μm filters and their mean cell size was 16%–46% smaller than that of the unfiltered community. FP consistently showed lower taxonomic richness than the 0.2RP fraction. Known small‐sized groups like Pelagibacterales, Actinobacteriota and Thermoplasmatota were significantly enriched in FP assemblages, but typical normal‐sized bacteria such as Flavobacteriales were also detected, suggesting the presence of miniaturized cells of widespread marine prokaryotes. Distinct environmental drivers shaped different FP subpopulations, as the contribution of specific taxa within the FP communities varied with organic carbon (OC) concentration and temperature. Notably, taxonomic dissimilarity between both fractions increased with higher OC concentration, suggesting that more normal‐sized taxa may be miniaturized towards conditions of less OC availability. Overall, our results suggest that the widespread use of 0.2‐μm filters likely results in significant losses of the extant diversity, potentially hiding ecologically important roles in marine ecosystems.

## 1 Introduction

Microorganisms passing through 0.2‐μm pore‐size filters, commonly referred to as ultramicrobacteria, have been observed for several decades in aquatic environments including marine and estuarine waters (Li and Dickie 1985; Hood and MacDonell 1987; Little et al. 1987; Gasol and Morán 1999; Vybiral et al. 1999; Elsaied et al. 2001; Velimirov 2001; Lannes et al. 2019, 2021; Obayashi and Suzuki 2019), freshwater systems (Hahn et al. 2003; Hahn 2004; Wang et al. 2007; Maejima et al. 2018; Park et al. 2023) or groundwater (Miyoshi et al. 2005; Luef et al. 2015; Herrmann et al. 2019). Such ‘filterable’ prokaryotes (hereafter FP) have been suggested to comprise different types of cells, including (i) miniaturized cells of taxa that reduce their size as a survival strategy to persist under unfavourable conditions, but which may become large again if conditions change; (ii) truly minute prokaryotic groups that do not increase their size regardless of favourable external conditions and (iii) normal‐sized prokaryotes able to squeeze through the filters due to higher flexibility or elongated shapes with thinner diameters (Ghuneim et al. 2018; Nakai 2020), and refs. therein. However, 0.2‐μm pore‐sized filters are still widely used for prokaryotic biomass collection in genetic or microscopic analyses. Consequently, most aquatic prokaryote surveys may have consistently disregarded a potentially relevant fraction of the extant diversity, leading to biases in our understanding of the identity, distribution, drivers and role of microorganisms in aquatic ecosystems.

Several studies have indicated the presence of both true ultra‐small cells (hereafter considered ‘ultramicrobacteria’) and potential starvation forms in 0.2‐μm‐filtered seawater samples. Early studies using clone libraries or DGGE showed that FP comprised mostly typical marine groups belonging to Gammaproteobacteria, Alphaproteobacteria and Bacteroidota, which supports the presence of miniaturized starvation forms of these groups (MacDonell and Hood 1982; Elsaied et al. 2001; Haller et al. 2000; Obayashi and Suzuki 2019). This type of FP could be locally inactive but might represent a fraction of the reservoir of diversity able to reactivate upon changing conditions (the microbial ‘seed bank’ (Lennon and Jones 2011); Pedrós‐Alió 2006) and hence they may play relevant roles in ecosystems. For example, 0.2‐μm FP from Japan, the Caribbean and the Sargasso Sea showed high growth potential, extracellular protease activity, or high amino acid uptake during experimental incubations (Li and Dickie 1985; Obayashi and Suzuki 2019), supporting that FP can reactivate if conditions change. However, all these studies used molecular approaches that identify only the most abundant taxa within communities, so it is possible that they missed a fraction of the extant FP diversity. Further, none of them explored the drivers or the spatio‐temporal variations of this FP cell pool. Hence, the extent to which filtration through 0.2 μm captures comparable or distinct diversity pools across space or time remains unknown.

As a general definition, true or obligate ultra‐small prokaryotes (less than 0.1 μm^3^ in cell volume) are considered to retain a small cell volume even when they are actively growing (Velimirov 2001; Duda et al. 2012). Some small marine bacteria such as Candidatus *Actinomarina minuta* (Ghai et al. 2013) and members of the SAR11 clade (e.g., *Pelagibacter ubique*; Rappe et al. 2002; Giovannoni 2017) are considered true marine ultramicrobacteria, but these groups are also commonly retrieved onto 0.2‐μm pore‐size filters, so they likely do not contribute much to the overlooked FP diversity. More recently, an enormous diversity of even smaller prokaryotes has been discovered: Patescibacteria (or Candidate Phyla Radiation, CPR), as well as the DPANN archaea (Diapherotrites, Parvarchaeota, Aenigmarchaeota, Nanoarchaeota and Nanohalorchaeota (Rinke et al. 2013; Brown et al. 2015; Hug et al. 2015; Luef et al. 2015)). These are enigmatic groups with unusual characteristics such as very small size (0.009 ± 0.002 μm^3^; Luef et al. 2015) as well as reduced genomes lacking common biosynthetic capabilities. In the ocean, some Patescibacterial groups have been detected in the 0.2‐μm filtrate in deep‐sea hydrothermal fluid using clone libraries (Naganuma et al. 2007), but also in the larger 0.2–3 μm and > 3 μm size‐fractions in North Sea surface seawater through 16S rRNA amplicon sequencing (Rahlff et al. 2020). More recently, the application of metagenomics to the < 0.2‐μm size fraction collected during the global Tara Oceans expedition showed enrichment in CPR, DPANN and taxonomically unclassified sequences, which appear to harbour a diversity of metabolisms related to carbon fixation and methane, nitrogen and sulfur cycling (Lannes et al. 2019, 2021). This implies that cells escaping common sampling strategies may hide a significant share of the marine functional diversity. Given that a shift towards smaller cell sizes has already been shown in heterotrophic bacteria and archaea as a likely consequence of ocean warming (Morán et al. 2015), it is possible that the relevance of ultra‐small prokaryotes in the ocean could increase in coming years.

Here we aimed at exploring the taxonomic composition of FP across contrasting marine sites to test whether this pool of prokaryotes varies spatially or along environmental gradients. In particular, we wanted to explore whether gradients in factors such as organic carbon or temperature might explain changes in the FP fraction. We quantified prokaryotic abundance, cell size and characterized the diversity of prokaryotic communities from two size‐fractions: (i) larger than 0.22‐μm (hereafter 0.2RP) and (ii) smaller than 0.22‐μm collected onto 0.1‐μm pore‐sized filters (FP). The study was conducted on six samples from three environmentally different marine regions: the Bay of Biscay (N Atlantic), the Mediterranean Sea and the Red Sea. In the latter basin, we also included an assessment of the temporal variability of the FP and 0.2RP pools at the same site by sampling on three different occasions.

## 2 Materials and Methods

### 2.1 Sampling and Basic Parameters

Surface seawater samples were collected from four different marine sites (Figure S1): (i) Med_BBMO, the Blanes Bay Microbial Observatory (BBMO), an oligotrophic coastal site in the NW Mediterranean that has been sampled monthly since 2001 (Gasol et al. 2012); (ii) Atl_D2, the D2 coastal station (110 m depth) that forms part of a coastal observatory with monthly samples collected by AZTI since 2014; (iii) Atl_G2, a mid‐shelf station (110 m depth) that forms part of the IEO RADIALES‐RADCAN time series of Gijón‐Xixón and has been sampled on a monthly basis since 2001 (Morán et al. 2015); and (iv) Red, a station (*ca*. 700 m depth) located off King Abdullah Economic City (KAEC) in the Saudi Arabian coast of the central Red Sea (Al‐Otaibi et al. 2020). Each site was sampled only once in late summer (Sept or Oct 2019, Table 1), except for the Red Sea, which was sampled on three different occasions representing summer (Sept and Oct 2019) and winter (Feb 2020), so‐called Red_Sep, Red_Oct and Red_Feb, respectively (Table 1).

**TABLE 1:** Location and date of the six samplings conducted, and measured physicochemical parameters.

	Med_BBMO	Atl_D2	Atl_G2	Red_Sep	Red_Oct	Red_Feb
Location	Blanes Bay (NW Med)	Bay of Biscay (Stn. D2)	Bay of Biskay (Stn. G2)	Red Sea	Red Sea	Red Sea
Lat (°)	41.670	43.367	43.675	22.472	22.472	22.472
Long (°)	2.800	−1.926	−5.578	39.035	39.035	39.035
Date	2019‐09‐17	2019‐09‐18	2019‐10‐03	2019‐09‐24	2019‐10‐22	2020‐02‐10
Temp (°C)	24	21.4	18.8	31.2	31.7	24.3
Sal	38.11	34.89	35.40	39.70	39.75	39.69
TOC (μM)	81.2	93.3	74.8	77.7	61.5	65.6
TN (μM)	3.6	5.1	3.3	8.3	6.2	4.2
Silicate (μM)	51.5	36.7	34.7	58.8	45	108.9
Nitrite (μM)	7.3	0.3	0.7	1.6	0.4	0.2
Nitrate (μM)	37.1	13.2	n.d.	5.9	0.6	6.8
Phosphate (μM)	0.5	1.1	0.3	n.d.	2.4	4.3
Chl (μg L^−1^)	0.28	0.22	0.16	0.10	0.13	0.27

*Note:* TOC, TN and inorganic nutrient concentrations are expressed in μmol L^−1^.

Abbreviations: Chl, chlorophyll a concentration; n.d., not detected; Sal, salinity; Temp, Temperature; TN, total nitrogen; TOC, total organic carbon.

Surface (0.5 m) seawater from each location was collected in duplicate or triplicate 10‐L acid‐washed polycarbonate carboys from a small boat and was carefully transported within 1–2 h to the respective laboratories, where it was immediately processed as described below. At each site, temperature and salinity were measured in situ using a CTD SeaBird 25 Profiler.

Samples for inorganic nutrients (nitrate, nitrite, phosphate and silicate) were kept frozen until further analyses with commercial segmented flow analysers: Alliance Evolution II (Med_BBMO samples), Bran‐Luebbe (Atl_D2 samples) and Seal Analytical (Atl_G2 and Red samples). Total organic carbon (TOC) and total nitrogen (TN) were measured by high temperature catalytic oxidation (HTCO) with a Shimadzu TOC‐L analyser. Chlorophyll a (Chl a) was extracted in 90% acetone and measured by fluorometry (Turner Designs fluorometer) from 150 mL of seawater filtered through GF/F (Whatman).

### 2.2 Prokaryotic Abundance and Cell Size

Samples for determining prokaryotic abundance and cell size were analysed with a FACSCanto II flow cytometer (BD Biosciences) as explained in Gasol and Morán (2015). Right angle light scatter signals normalized by the value of 1 μm fluorescent beads added to each sample were used for estimating the mean diameter of prokaryotic cells (Calvo‐Díaz and Morán 2006). Cell size estimates are given as volumes (μm^3^), assuming spherical shape.

### 2.3 DNA Extraction and SSU rRNA Metabarcoding of Prokaryotic Communities

Between 1 and 10 L of water were sequentially filtered through 0.22‐μm and 0.1‐μm polyethersulfone (PES) membrane filters (47 mm, Linkman Group) in duplicates or triplicates (Table S1) for molecular analysis. Each bottle was considered a replicate and was filtered separately from the rest. The collected size‐fractions represented the commonly retrieved free‐living cell fraction (0.2RP, i.e., “0.2‐um retained prokaryotes”) as well as the filterable prokaryotes (FP). In Blanes Bay (Med_BBMO), an additional replicate was used to sequentially filter 1 L through 0.2 and 0.1 μm to visually verify the retention of cells onto the 0.1 μm filter. A section of each filter was cut and stained with DAPI (4′,6‐diamidino‐2‐phenylindole) for their observation under the epifluorescence microscope (ZEISS Axio Imager).

A total of 34 filters were collected and immediately stored at 80°C until further processing. The DNA was extracted from the frozen filters using the DNeasy PowerWater Kit (Qiagen), following manufacturer's instructions and the amount of extracted DNA was quantified using the Qubit 2.0 Fluorimeter (Invitrogen) and the Qubit DNA High Sensitivity (HS) assay. 12.5 ng of DNA were used as template for the preparation of Illumina MiSeq libraries using primers 515F‐Y and 926R (Parada et al. 2016) to amplify the hypervariable V4‐V5 regions of the 16S rRNA the and Nextera XT DNA Library Preparation Kit (Illumina), following the standard procedure for the preparation of 16S Metagenomic Sequencing Libraries (Illumina). Sequencing was carried out at the Bioscience Core Lab at KAUST (corelabs.kaust.edu.sa/labs/bioscience‐core‐lab).

### 2.4 Sequence Data Analysis

Raw SSU (16S) rRNA amplicon sequence data was processed as previously described by Lanzén et al. (2021). Briefly, read‐pair overlapping and initial filtering was carried out using *vsearch* v2.7.1 (Rognes et al. 2016) allowing up to 20 mismatches, and primers removed using *cutadapt* v1.18 (Martin 2011) followed by truncation to 369 bp. Reads with incorrect primer sequences (more than one mismatch) or with more than one expected error were discarded. The remaining sequences were de‐replicated, sorted by abundance and subjected to iterative clustering using Swarm v2.21 (Mahé et al. 2015) into sequence variants (SVs). An SV contingency table based on Swarm output was derived using the scripts *fasta_merging.py* and *matrix_creation.py*, available from SLIM (Dufresne et al. 2019). Singletons and putative chimeras were identified using vsearch (reference based with SilvaModPR2 v138 as sequence database (Lanzén et al. 2012; https://github.com/lanzen/CREST) and followed by de novo) and discarded. To correct for remaining artefacts and to merge intra‐specific or intra‐genomic SVs, we then applied LULU post‐clustering curation (Frøslev et al. 2017) to merge SVs with highly correlated co‐occurrence patterns, sharing at least 97% sequence similarity.

Curated SVs were aligned to SilvaModPR2 v138 with blastn v2.6.0+ and taxonomically classified using CREST v3.1.0 (https://github.com/lanzen/CREST; Lanzén et al. 2012). SVs originating from plastid, mitochondrial or eukaryotic rRNA were removed, as well as sequences that could not be classified to at least phylum rank. Cross‐contamination was reduced by setting SV abundances to zero where it occurred in a sample at very low abundances compared to its average abundance across samples (< 1%). Finally, to compensate for differences in sequencing depth, all SVs that did not reach a relative abundance of at least 0.02% in any sample were removed (corresponding to 4 reads in the sample with the lowest depth after filtering). For taxonomic richness estimates, the SV table was rarefied to the minimum number of reads per sample (71,801). Raw sequence data are publicly available in the Sequence Read Archive (www.ncbi.nlm.nih.gov/sra) under accession number (PRJEB86314). The abundance‐filtered non‐rarefied SV table, taxonomy table and environmental data used in this study table are provided as Tables S4–, S6.

Functional prediction was carried out using Tax4Fun2 v1.1.5 (Wemheuer et al. 2020) based on alignment of the abundance filtered SV table to the Ref99NR database (minimum identity 97% and normalized by copy number). In order to look for biogeochemically relevant genes, we followed the selection of 20 key functional genes made by Auladell et al. (2023). We first queried our annotation table by gene names as reported in their study and, for each match, retrieved the corresponding KEGG Ortholog (KO) present in our Tax4Fun2 outputs. Because Auladell et al. (2023) combined KO‐based HMMs (KOfam) with COG‐based reverse PSI‐BLAST for phosphorus‐related genes and MicRhoDE for proteorhodopsins (i.e., not all targets were specified by KOs) we mapped gene names to KOs when an explicit KO existed, recovering 16 of the 20 target genes proposed by Auladell et al. (2023) in our KO set. The tables containing KO abundance and descriptions are provided as Tables S7 and S8.

### 2.5 Identification of SVs With Preference for a Give Size‐Fraction

In order to explore whether each size‐fraction preferentially recovered different taxonomic groups, we defined an index of size‐fraction preference (analogous to the PAN‐index presented in Salazar et al. (2015) used to distinguish between free‐living and particle‐attached taxa). This index indicates in which size‐fraction an SV is more abundant and was calculated using the abundance‐weighted mean of each SV between the two size‐fractions. FP samples were given a value of 1, and 0.2RP samples a value of 2, so that SVs occurring only in FP samples would have an index value of 1, SVs exclusively present in 0.2 µm filters would have a value of 2, and SVs equally distributed across both size‐fractions would have a value of 1.5. The distribution of this size‐fraction preference index values showed two peaks in 1 and 2 (see Figure 4a), so we differentiated SVs enriched in small size‐fractions (index ≤ 1.2) from those enriched in large size‐fractions (index ≥ 1.8). SVs with intermediate values were considered as having no clear preference for any of the two size‐fractions.

### 2.6 Statistical Analyses

Differences between prokaryotic communities were visualized using non‐metric multidimensional scaling (NMDS) using the metaMDS function of the R vegan package (Oksanen et al. 2015) based on Bray‐Curtis distances. The environmental variables were fitted onto the NMDS ordination using the envfit function, and only variables showing significant correlations (*p* < 0.05) were retained and displayed as vectors in the NMDS plot. Differences in taxonomic composition among size‐fractions or sites were tested using analysis of similarity (ANOSIM R Vegan). Prokaryotic taxonomic richness (i.e., number of SVs per sample) and diversity (Shannon index) were calculated using the rarefied SV table. Spearman correlations were used to explore the variation in microbial parameters in relation to physicochemical variables (R ggpubr) and the results of the correlation analysis were presented in a heat‐map (R ComplexHeatmap). Statistical differences in KO abundance between size fractions were tested using Wilcoxon rank‐sum test (stats R package). All analyses were run using R software version 2022.12.0 (R Core Team 2018).

## 3 Results

### 3.1 Physicochemical Conditions

Temperature, salinity, TOC, TN and inorganic nutrient concentrations varied largely between the studied sites (Table 1). Water temperature was relatively high during all samplings, ranging between 21°C in Blanes (Med_BBMO) to 32°C in the Red Sea in summer (although in winter ‐Red_Feb‐ temperature in the Red Sea decreased to 24°C). Salinity varied from 34.9 in Atl_D2 to *ca*. 39.7 in the three Red Sea samplings. Total organic carbon was lowest in the Red_Oct (61 μmol L^−1^) and highest in Atl_D2 (93 μmol L^−1^), while total nitrogen ranged from 3.3 to 8.3 μmol L^−1^. Nitrate and nitrite concentrations were highest in the Mediterranean, whereas phosphate and silicate were maximum during the winter Red Sea sampling (Red_Feb). Chl*a* was generally low, ranging between 0.10 and 0.28 μg L^−1^ (Table 1).

### 3.2 Variation in Prokaryotic Abundances and Cell Size Across Size‐Fractions

**FIGURE 1:**
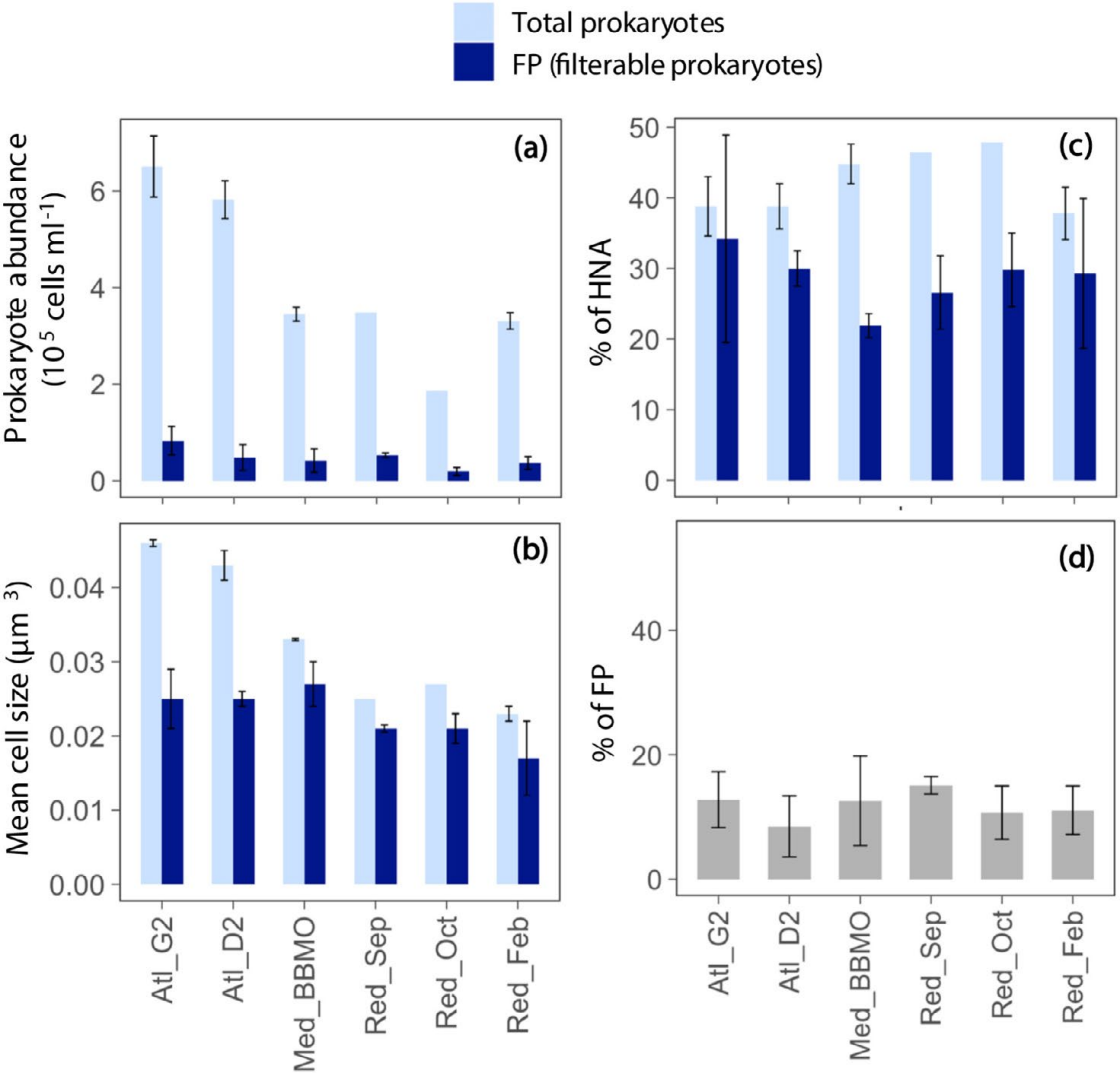
Abundance (a) and estimated mean cell size (b) of total prokaryotes and of cells passing the 0.22 μm filter (i.e., FP) in each sampling. Percentage of HNA cells in both total and FP populations, (c) and proportion of FP cells (shown as % of total prokaryotes, d) across samplings. Shown are means and standard deviation of replicates.

Total abundances of heterotrophic prokaryotes (i.e., including also FP cells) ranged from 1.87 to 6.51 × 10^5^ cells mL^−1^ with 8.5%–15.1% of total cells belonging in the FP fraction (Figure 1a,d). Mean cell size (considering total prokaryotes) varied between 0.023 and 0.043 μm^3^ (Figure 1b), and was 19% and 84% larger than in FP (0.017–0.027 μm^3^). Both abundance and size were highest at the Bay of Biscay sites and lowest in the Red Sea. The size difference between both populations was highest in Atl samples, where FP populations were 42% (Atl_D2) and 46% (Atl_G2) smaller than total prokaryotes. In the remaining cases, the average size reduction was less than 26%. The proportion of prokaryotic cells with high nucleic acids content (HNA cells), typically larger cells and usually comprising copiotrophic bacterial taxa (Vila‐Costa et al. 2012), varied from 38% to 49% of total cells (Figure 1c). In the FP fraction, lower percentages of HNA (22%–34%) were recovered (Figure 1c).

### 3.3 Taxonomic Richness and Composition of 0.2RP Versus FP Communities

**FIGURE 2:**
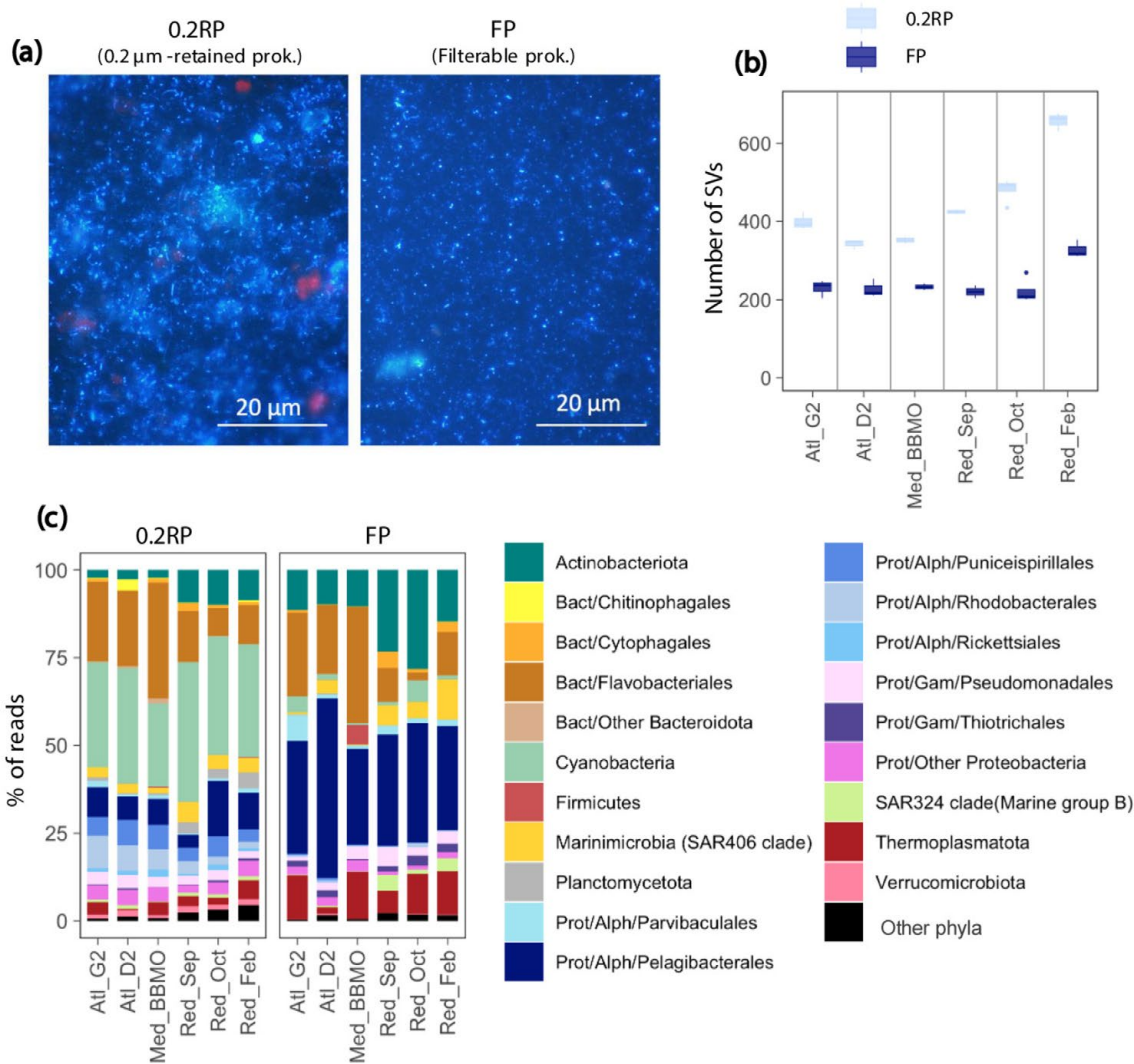
(a) Micrographs of microorganisms retained onto the 0.2 and 0.1 μm pore‐sized filters after sequential filtration of 1 L of seawater from Med_BBMO. (b) Number of SVs per site, distinguishing between both size‐fractions. (c) Taxonomic composition of the communities from both size‐fractions. The classification was performed at the phylum level except in some cases where either classes or orders were distinguished. Bact, Bacteroidetes; Prot, Proteobacteria. Most phyla comprising < 0.6% of the total sequences were grouped as ‘Other Phyla’ except for Firmicutes to highlight its local increase in relative abundance in Med_BBMO.

Sequential filtration of seawater through 0.2 and 0.1 μm membranes enabled the separation of 0.2RP and FP communities for subsequent characterization by DNA sequencing. Micrographs of both filter types reveal a substantial number of visibly small organisms, along with some larger cells after filtering just 1 L of seawater from Blanes (Figure 2a).

A total of 5,634,102 quality‐checked sequence reads were retrieved from the 34 samples, which clustered into 1881 SVs. After rarefaction, 1800 SVs (2,441,234 reads) remained. The SV number per sample ranged between 201 and 677 and was consistently higher in the 0.2RP (mean ± SD = 452 ± 112 SVs) than in the FP size‐fraction (244 ± 45, Figure 2b). Communities from the winter Red Sea sampling (Red_Feb) showed the highest richness in both fractions. The largest difference in SV number between fractions was also found in Red Sea samples, in which 0.2RP harboured up to 350 more SVs (2.2‐fold more) than FP assemblages (Figure 2b). Communities from Atl_D2 and Med_BBMO displayed the smallest difference in richness between size‐fractions.

Considering all samples, 32 prokaryotic phyla were identified (Figure 2c). All of them were found in the 0.2RP communities, which were dominated by Cyanobacteria (32% of total reads), Proteobacteria (29%), Bacteroidota (18%), Actinobacteriota (6.2%), Marinimicrobia (SAR406 clade, 3.5%), Thermoplasmatota (2.7%), Planctomycetota (2%) and Verrucomicrobiota (1%). A lower number of phyla (*n* = 19) was retrieved in FP assemblages, which were similarly dominated by Proteobacteria (45%), Bacteroidota (18%), Actinobacteriota (16%), Thermoplasmatota (10%), Marinimicrobia (SAR406 clade, 3.5%) and Cyanobacteria (2.7%).

The taxonomic composition of both prokaryotic fractions was relatively consistent across the studied sites, at least at the phylum or class level, with 0.2RP‐communities being dominated by Cyanobacteria (mostly Synechococcales), Flavobacteriales and lower proportions of Actinobacteriota and Alpha‐ and Gammaproteobacterial groups (Figure 2c, Figure S2). In comparison, FP communities showed higher proportions of the Alphaproteobacteria Pelagibacterales and of the phyla Actinobacteriota (mostly Candidatus *Actinomarina*) and Thermoplasmatota (mostly Marine Group II), and much lower proportions of Cyanobacteria, Planctomycetota and Verrucomicrobiota. Within Proteobacteria, Parvibaculales (Alphaproteobacteria) and Thiotrichales (Gammaproteobacteria) increased their relative abundances in FP communities, whereas Puniceispirillales, Rhodobacterales and Rickettsiales (Alphaproteobacteria) decreased (Figure 2c, Figure S2).

**FIGURE 3:**
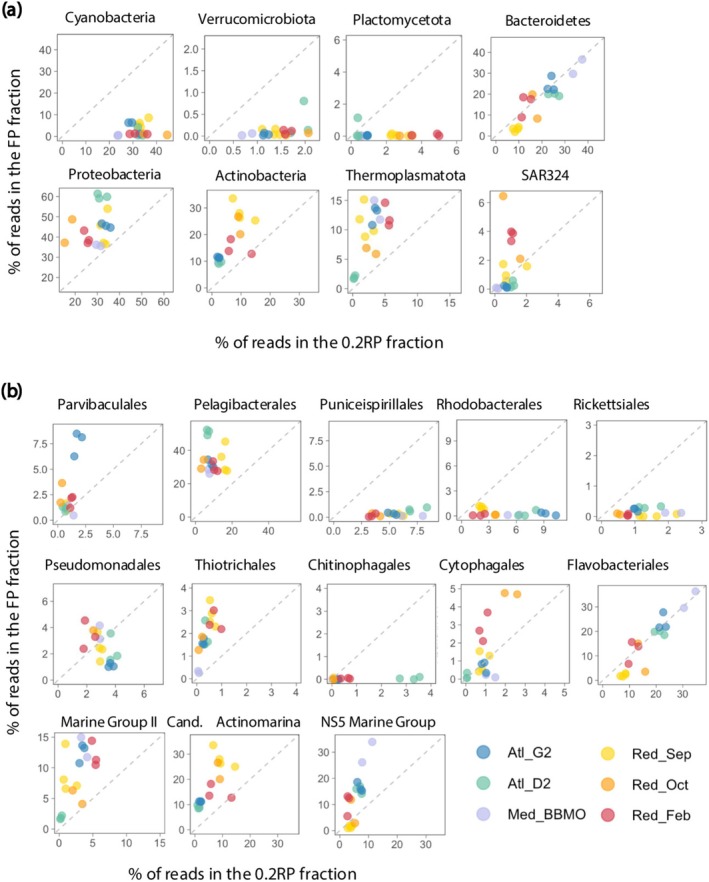
(a) Comparison of the relative abundances of the dominant phyla between the two size‐fractions, colour‐coded by sampling. (b) Comparison of the relative abundance between the two size‐fractions of the most abundant orders within Alphaproteobacteria (Parvibaculales, Pelagibacterales, Puniceispirillales, Rhodobacterales, Rickettsiales), Gammaproteobacteria (Pseudomonadales, Thiotrichales) and Bacteroidetes (Chitinophagales, Cytophagales, Flavobacteriales), as well as three of the dominant groups within FP communities (Marine Group II, Thermolasmatota; Cand. *Actinomarina*, Actinobacteriota; NS5 marine group, Flavobacteriales). Colours indicate the different sampling sites and dates. Each dot represents an individual replicate, and the dashed line indicates the 1:1 line.

For most prokaryotic groups, the size‐fraction driven changes in relative abundance were fairly consistent across all sites (Figure 3). For example, Cyanobacteria, Planctomycetota and Verrucomicrobiota were always more abundant in the 0.2‐μm‐size‐fraction, whereas Proteobacteria (mostly Pelagibacterales), Actinobacteria (mostly Cand. Actinomarina) and Thermoplasmatota (mostly Marine Group II) showed consistently higher relative abundances in the FP fraction in all cases (Figure 3a,b). Only Bacteroidetes (and Flavobacteriales within them, Figure 3) showed similar contributions in both size‐fractions, but FP communities were significantly enriched in the Flavobacterial NS5 marine group in most cases (Figure 3, Figure S2).

### 3.4 Size‐Fraction Preference of SVs

Out of the 1881 SVs, 766 (41%) could only be detected in the 0.2RP fraction (in either one or more 0.2RP samples), whereas only 173 (9%) were unique to the FP fraction. However, most unique SVs were rare, as they never accounted for more than 6% or 2% of the 0.2RP and PF sequences, respectively (Table S2). Consequently, the majority of the sequences in both fractions belonged to SVs that were shared between the two.

**FIGURE 4:**
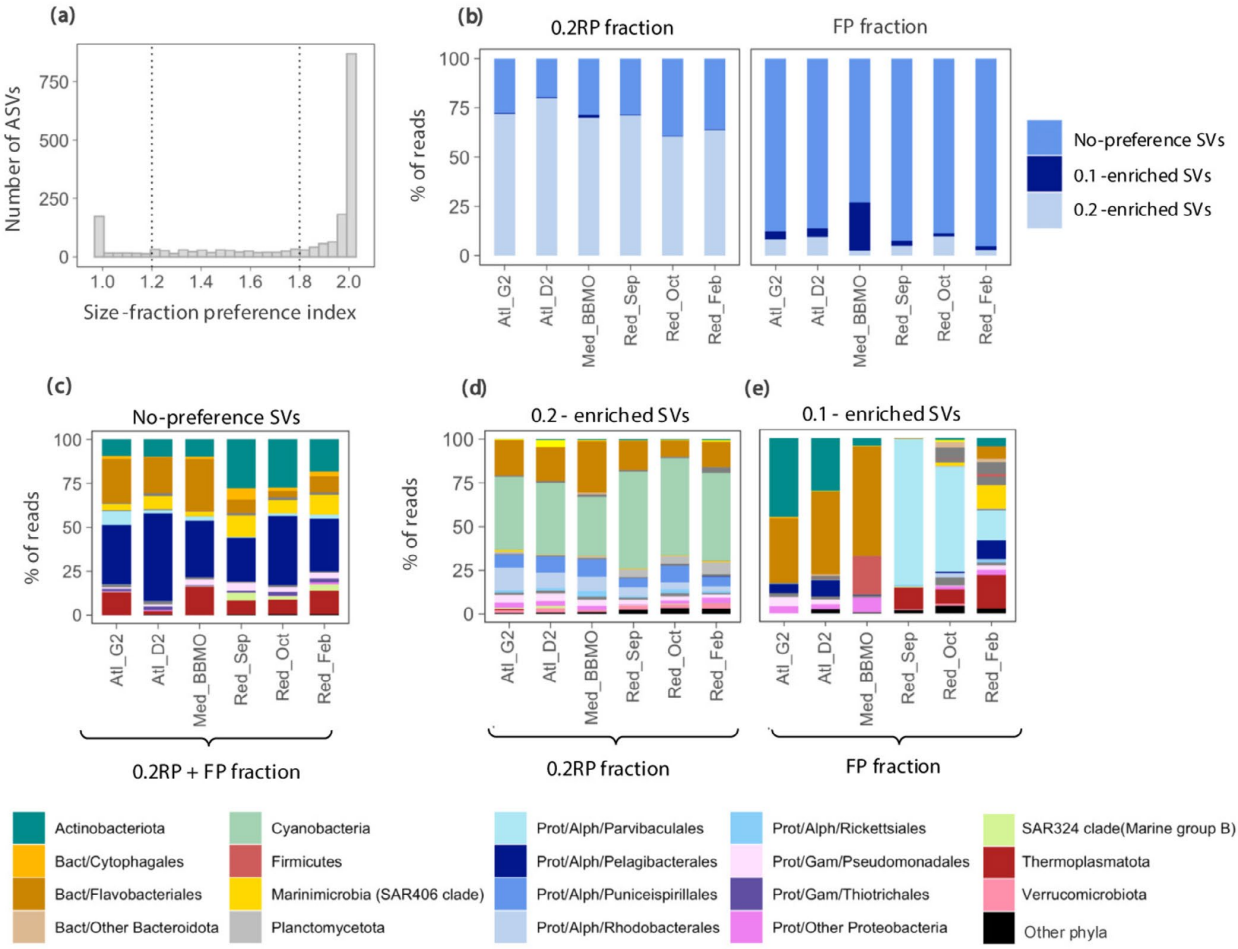
(a) Distribution of the size‐fraction preference index values for all SVs (see Methods). The dotted lines indicate the values we took for distinguishing SVs with preference for the small‐size‐fraction (Index ≤ 1.2, ‘0.1‐enriched SVs’, *n* = 254) and those mainly enriched in the large size‐fraction (Index ≥ 1.8, ‘0.2‐enriched SVs’, *n* = 1244). Those in between were considered as SVs with no clear preference for a given size‐fraction (*n* = 383, no‐preference SVs). (b) Contribution (% of sequences) of the three groups of SVs to the studied communities. (c–e) Taxonomic composition of the three groups of SVs across samplings, showing: (c) no‐preference SVs on the entire dataset; (d) 0.2‐enriched SVs in > 0.22 μm‐samples; and (e) 0.1‐enriched SVs in FP (0.22–0.1 μm) samples.

We hence explored whether certain taxa were preferentially captured by each pore‐size filter, regardless of whether they could be also detected in the other size fraction. To do so, we categorized SVs using an abundance‐weighted mean for each SV (see Materials and Methods). We found that the distribution of this size‐fraction preference index values showed two peaks in 1 and 2 (Figure 4a), so we differentiated SVs enriched in small size fractions (index ≤ 1.2, 0.1‐enriched SVs) from those enriched in large size fractions (index ≥ 1.8, 0.2‐enriched SVs). SVs with intermediate values were considered as having no clear preference for any of the two size fractions. A larger number of SVs was categorized as 0.2‐enriched (*n* = 1244 SVs) compared to those enriched in 0.1‐assemblages (254 SVs). 383 SVs did not show any clear preference. 0.2RP communities were dominated by 0.2‐enriched SVs (range 56%–81% of total sequences), whereas FP assemblages showed mostly taxa with no preference (71%–95%, Figure 4b). 0.1‐enriched SVs, instead, comprised < 5% across all sites except in Med_BBMO, where they accounted for 26% of FP assemblages (Figure 4b).

0.2‐enriched SVs comprised mostly Cyanobacteria, Flavobacteriales (Bacteroidota) and Punieispirillales and Rhodobacterales (Alphaproteobacteria) across all samplings (Figure 4d). Conversely, the composition of 0.1‐enriched SVs differed between those from the Atlantic and Mediterranean and those from the Red Sea (Figure 4e): Flavobacteriales and Actinobacteria (mostly Cand. Actinomarina) dominated Atl samples, Flavobacteriales and Firmicutes dominated Blanes samples and large proportions of Parvibaculales (mostly PS1 clade) and Thermoplastatota (Marine Group II) were found in Red Sea samples, the former largely dominating summer communities (Figure 4e, Figure S2). SVs with no size‐fraction preference, instead, consistently comprised Pelagibacterales and Thermoplasmatota, as well as Actinobacteriota, Flavobacteriales and Marinimicrobia (Figure 4c).

### 3.5 Size‐Fraction Differences in Predicted Functional Potential

**FIGURE 5:**
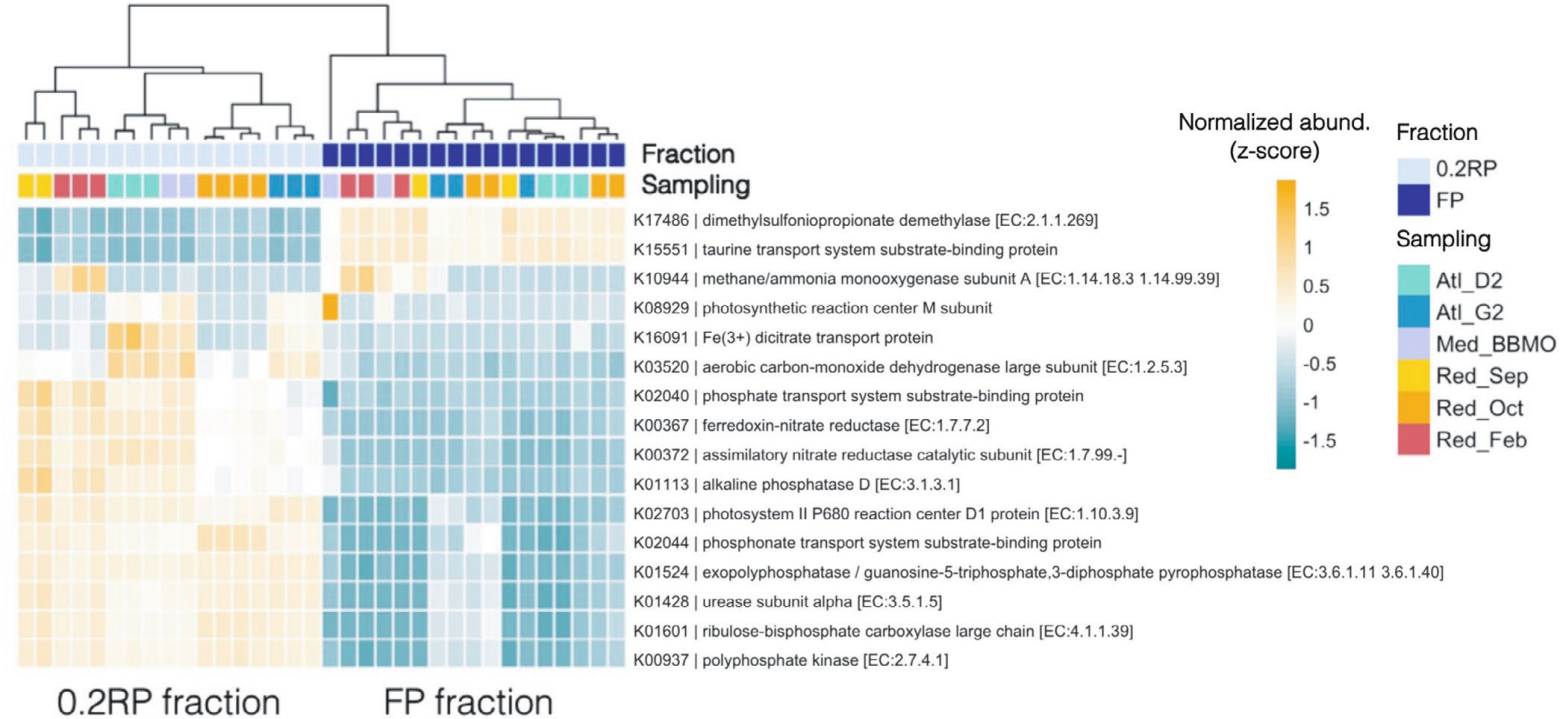
Heatmap showing the relative abundance patterns of the 16 biogeochemically significant KOs (following Auladell et al. 2023) across samples from each size‐fraction (0.2‐enriched vs. FP‐enriched). KO relative abundances were predicted from our ASVs using Tax4Fun2 (see Section 2), and were standardized across samples using *z*‐scores to highlight relative enrichment patterns. Rows represent KOs enriched in each fraction, and columns correspond to individual samples. Colour intensity reflects the deviation from the mean abundance of each KO (*z*‐score). Top colour bars indicate size fraction and sampling, respectively.

To assess whether these taxonomic differences translated into shifts in functional potential, we predicted KO profiles for all ASVs using Tax4Fun2. Most predicted KOs were shared between the two size fractions (*n* = 7845 out of 8083), while those unique to the FP fraction (*n* = 99) were extremely rare and not all associated with prokaryotes, likely reflecting noise or incidental contamination (details not shown). We therefore focused on the 20 genes previously identified by Auladell et al. (2023) as biogeochemically relevant in coastal marine systems, of which 16 were detected in our dataset. All but one KO (K10944, ferredoxin–NADP reductase subunit) showed significant differences between the two fractions (Wilcoxon rank‐sum tests, *p* = 0.003–0.0001; Figure 5, Figure S3). It must be noted, though, that because the representation of genomes belonging to the dominant groups is uneven, the predicted functional differences may be, at least partly, a direct reflection of the taxonomic differences observed among size fractions and therefore should not be considered independent functional evidence.

Only two KOs were significantly enriched in the FP fraction relative to the 0.2RP assemblages: K15551, encoding the taurine transport system substrate‐binding protein (*tauA*) and K17486 (*dmdA*), the dimethylsulfoniopropionate (DMSP) demethylase.

In contrast, several KOs were enriched in the 0.2RP communities, including K01601, coding for malate dehydrogenase involved in central carbon metabolism; K02703, part of the cytochrome c oxidase complex linked to aerobic respiration; K03520, a transcriptional regulator associated with stress‐responsive gene expression; K00367 and K00372, encoding nitrite reductase subunits involved in nitrogen transformations; and K01428, a glycosyl hydrolase participating in polysaccharide degradation. The > 0.2 μm fraction also showed higher abundances of K01524, a formate dehydrogenase subunit associated with C1 metabolism; K02044 and K02040, components of ABC‐type transport systems likely mediating substrate uptake in particle‐associated microbes; K00937, encoding acetyl‐CoA synthetase essential for acetate assimilation; K01113, a glucosidase involved in carbohydrate breakdown; K08929 (pufM, photosynthetic reaction centre M subunit); and K16091, a ferredoxin‐related oxidoreductase. These 16 KOs were visualized in a heatmap (Figure 5), unveiling highly consistent abundance patterns of most KOs across samples belonging to the same size fraction (Figure 5, Figure S3).

### 3.6 Environmental Drivers of Abundance, Size and FP Community Structure

In general, the abundance and cell size of both total prokaryotes and FP tended to increase with increasing DOC, and to decrease with increasing phosphate, silicate, salinity and temperature. However, the only significant relationships found were negative correlations between silicate and average cell size of total prokaryotes (Spearman's ρ = −0.94, *p* = 0.02), and between temperature and the size difference between the total and the FP cells (Spearman's ρ = −0.82, *p* = 0.045), meaning that in warmer waters, filtration by 0.22 μm resulted in larger differences in cell size between the two fractions (Figure S4). Regarding taxonomic richness, that of the 0.2RP fraction showed a decrease with increasing DOC (ρ = −0.88, *p* = 0.033), whereas FP richness increased with chlorophyll *a* concentration (ρ = 0.89, *p* = 0.033). However, the latter relationship was largely driven by a single sample.

**FIGURE 6:**
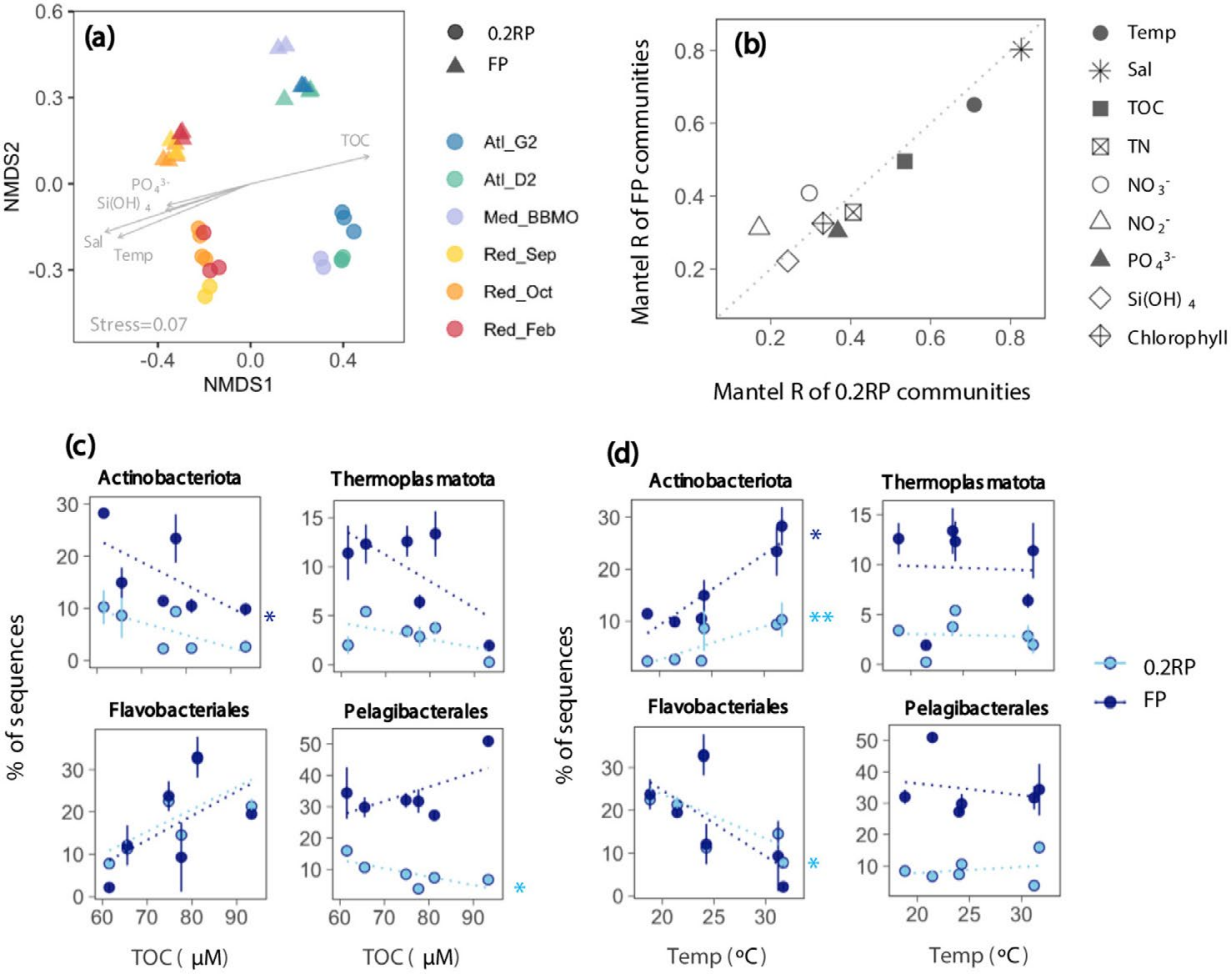
(a) Non‐multidimensional scaling analysis (NMDS) of all the studied microbial communities based on Bray‐Curtis dissimilarities. The two size‐fractions are indicated by symbols and colours indicate the different samplings. Environmental vectors fitted onto the NMDS ordination (envfit) indicate the most strongly correlated variables (*p* < 0.05). (b) Comparison of the R coefficients of the Mantel correlations (presented in Table S3) between variations in environmental variables and 0.2RP community dissimilarity versus the R coefficients of Mantel correlations performed with the FP communities. The symbols indicate the individual environmental variables considered in each Mantel correlation and the dotted line is the 1:1 line. (c, d) Correlations between the percentages of the four groups dominating FP communities along TOC (c) or temperature (d) gradients. The colour indicates the different size fractions, values are means and standard deviation of replicates. The asterisks indicate whether these relationships were significant (**p < 0.05, *p > 0.1. *n* = 6).

0.2RP communities differed clearly from FP assemblages in terms of their taxonomic structure in all six cases (Figure 6a, ANOSIM_byfraction_ = 0.63, *p* = 0.001). In addition, Red Sea communities clustered separately from the rest, while Atlantic and Mediterranean assemblages were more similar to each other (ANOSIM_bysampling_ *R* = 0.64, *p* = 0.001). The higher salinity, temperature and lower TOC of Red Sea samples appeared to explain this regional segregation of communities, as these variables showed the strongest significant correlations with the ordination (*p* < 0.05, envfit analysis).

Communities from the two size‐fractions appeared to be structured by the same drivers. In both cases, changes in salinity and temperature had the strongest correlations with community dissimilarities, followed by variability in TOC (Table S3). Indeed, both size‐fractions responded to site differences in the environmental variables in almost the same way, as the R coefficients of the respective Mantel correlations covaried strongly and positively (Figure 6b). TOC and temperature were the variables that appeared to explain some of the variation in the relative abundance of the FP dominant groups. Actinobacteria appeared to decrease in abundance with increasing TOC and decreasing temperature in both fractions, whereas Flavobacteriales showed the opposite pattern and behaved similarly in both size‐fractions (Figure 6c,d). On the contrary, Pelagibacterales showed opposite behaviours with TOC in each size‐fraction, increasing with higher TOC in the FP fraction and decreasing in the 0.2RP fraction (Figure 6c), potentially indicating different populations in each size fraction. Thermoplasmatota showed no clear patterns along these or other gradients.

**FIGURE 7:**
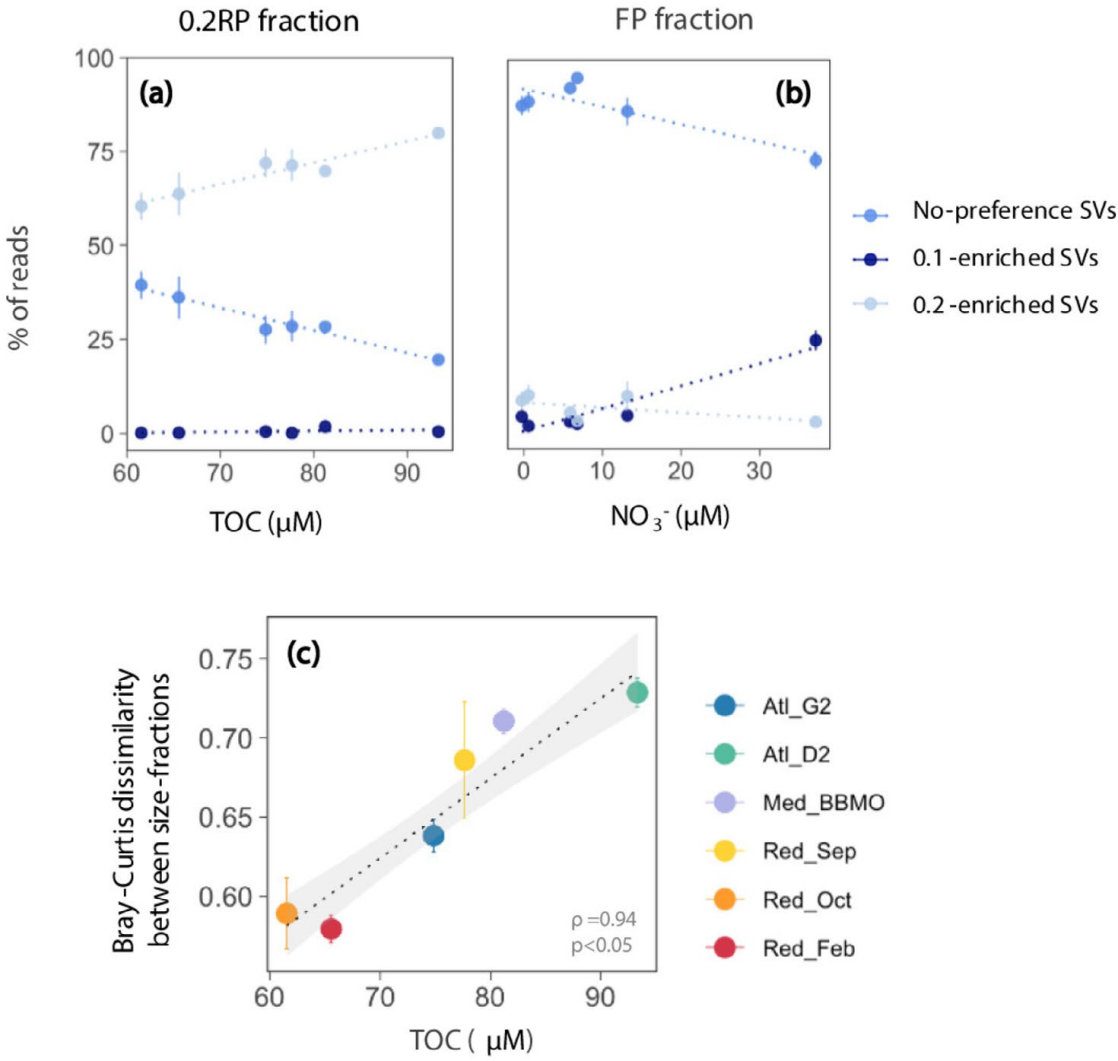
Variations in the contribution of the three categories of SVs (0.2‐enriched, 0.1‐enriched and no‐preference SVs) in 0.2RP communities along a gradient of TOC (a), or in FP communities along a gradient of nitrate (b). (c) Variation in Bray–Curtis dissimilarity between both size‐fractions along a gradient of TOC concentration (Spearman's rho = 0.94, *p* < 0.05, *n* = 6) (c). Values and error bars represent mean and standard deviation of replicates. Note that the three Red Sea (‘Red_’) samples represent the same station sampled on three different occasions.

We compared the relative abundance of the three SV categories with all the measured environmental properties. Considering 0.2RP communities, TOC was the variable most clearly (yet not significantly) correlated with an increased proportion of 0.2‐enriched SVs, which was accompanied by a decrease in the % of no‐preference SVs and no clear changes in 0.1‐enriched SVs (Figure 7a). Conversely, in FP communities, the only obvious pattern was an increased contribution of 0.1‐enriched SVs towards higher nitrate concentrations and the concomitant decrease of the other two groups (Figure 7b). Finally, we observed that the community dissimilarity between the two size‐fractions increased pronouncedly towards sites with higher TOC concentration (Figure 7c). This implies that the composition of FP communities was more similar to that of 0.2RP assemblages in sites with lower TOC, possibly reflecting a higher proportion of starvation forms of typical marine bacteria that could account for their presence in both fractions.

## 4 Discussion

### 4.1 A Non‐Negligible Fraction of Prokaryotes Is Lost Upon 0.2‐μm Filtration in Different Marine Sites

Despite repeated and clear evidence of bacteria passing through 0.22‐μm filters, few studies have quantified this diversity loss across different sites and this pore size is still widely used in aquatic prokaryotic studies involving genetic analyses or microscopy. Here, using polyethersulfone (PES) 0.22‐filters (Linkman), we observed that between 9% and 15% of total cells were lost upon filtration regardless of the in situ differences in prokaryotic abundance and cell size. We are aware that the retention capacity may vary largely between filters of the same theoretical pore size made of different materials, so it is possible that the FP proportion would change if using different filter types. For example, Gasol and Morán (1999) found differences between the mean percentage of cells passing 0.2‐μm‐pore‐size filters made of polycarbonate (range 2%–23% of total cells), inorganic aluminium polymers (18%–20%) and mixed cellulose esters (0.7%–8%). The proportion of cells missed with our PES filters falls within the range reported by these authors and also by Velimirov et al. (1992), who found that bacteria passing 0.2‐μm filters in seawater samples from the Western Mediterranean Sea varied from 4% to 11% of the total bacteria. The variability observed in ours and those previous studies suggests that the fraction of cells missed may vary substantially depending on the type of filter used and/or on additional environmental conditions or microbial features. Indeed, spatial changes in these percentages were attributed to smaller average bacterial sizes and different degree of attachment of bacteria to particles towards more oligotrophic open ocean waters (Gasol and Morán 1999). In our case, though, we could not relate the observed variations in FP proportion to any of the measured environmental parameters, nor to differences in cell abundance or size.

Cells passing our 0.2 μm‐filter had an estimated average size ranging from 0.017 to 0.027 μm^3^, which is considerably smaller than that of the low nucleic acid content (LNA) bacterial populations commonly reported from marine waters (e.g., 0.050–0.056 μm^3^ (Morán et al. 2015) or 0.035–0.06 μm^3^ (Huete‐Stauffer et al. 2016)), and slightly larger than the size reported for known ultra‐small marine bacteria such as SAR11 (0.037 ± 0.011 μm^3^; Zhao et al. 2017) and Cand. *Actinomarina minuta* (0.013 μm^3^; Ghai et al. 2013), two groups that were enriched in our < 0.2 μm size fractions. FP cells tended to be larger in waters with higher TOC and nitrite (Figure S4), and smaller in warmer and more saline waters, but these relationships were not significant. In any case, it seems safe to assume that most genetic or microscopy studies using 0.2 μm filters may have lost a potentially significant fraction of the extant prokaryotic biomass.

### 4.2 Filterable Prokaryotes May Include Both Small‐Sized Groups and Potential Starvation Forms

FP differed from 0.2RP communities in all studied sites, both in terms of richness and taxonomic composition. The four most abundant groups in FP communities were Pelagibacterales, the Actinobacteria Candidatus *Actinomarina*, the NS5 marine group (Flavobacteria) and the archaea Marine Group II (Thermoplasmatota), which together accounted for ca. 70% of the FP sequences. FP assemblages had consistently lower richness than 0.2RP. Despite these differences, most detected SVs were shared between the two size fractions, indicating that few SVs were exclusive to either fraction. This makes sense as filtration does not allow to unequivocally separate cells of different sizes. For instance, small cells may be retained in the large‐size fraction (0.2 μm) if the filter is clogged, whereas some large bacteria are sufficiently flexible to pass through the filters (Nakai 2020). Moreover, holes larger than the nominal pore size are not unfrequent in filters of different materials (Figure S5). We thus categorized SVs based on their prevalence on one fraction or the other using an abundance weighted mean analysis as in Salazar et al. (2015). Doing this, we found that the SVs that were enriched in FP communities (0.1‐enriched SVs) represented a small fraction of FP communities (range 5%–26% of reads). However, they differed between the Atlantic and Mediterreanean sites, which comprised mostly Flavobacteriales, Actinobacteria and/or Firmicutes and the Red Sea samples, where groups like Parvibaculales, Themoplasmatota or Marinimicrobiota were detected. Within the three Red Sea samples, moreover, the identity of these 0.1‐enriched SVs seemed to change seasonally, and the richness of both size fractions increased in winter, in accordance with previous findinds focusing on the 0.2RP in this area (García et al. 2015). However, longer‐term studies are required to properly address the temporal patterns of FP communities in marine systems.

FP communities were instead numerically dominated by SVs showing no clear preference for one size fraction (no‐preference SVs), belonging mostly to Pelagibacterales, Thermoplasmatota, Flavobacteriales and Actinobacteriota, and their contribution was relatively consistent across sites. These diversity patterns contrast with those found in previous studies exploring FP taxa in estuarine or marine waters (including the oligotrophic NW Mediterranean Sea and highly productive coastal waters in Japan or Alabama), in which a dominance of typical copiotrophic groups within Alpha‐ and Gammaproteobacteira (such as *Pseudomonas*, *Aeromonas*, *Alteromonas* or *Roseobacter*) was found (MacDonell and Hood 1982; Hood and MacDonell 1987; Vybiral et al. 1999; Haller et al. 2000; Obayashi and Suzuki 2019). These discrepancies may be due to the fact that all previous studies used either DGGE or clone libraries, which fail to capture a large fraction of the diversity, to actual community shifts in FP communities from different sites or moments, or to the use of different types of filters, as discussed above.

Members of the Pelagibacterales, Candidatus *Actinomarina* and Marine Group II have all been previously shown to have small cell sizes and streamlined genomes adapted to oligotrophic ocean conditions (Giovannoni et al. 2005; Ghai et al. 2013; Martin‐Cuadrado et al. 2015; Zhao et al. 2017). These three groups are also recognized as major contributors to ocean biogeochemistry. The global dominance of Pelagibacterales, for example, is linked to their capacity to scavenge extremely low concentrations of dissolved organic carbon compounds (Giovannoni 2017), and many harbour proteorhodopsins that enhance energy yields under carbon‐limited conditions (Giovannoni et al. 2005), supporting their persistence in the oligotrophic ocean. *Ca*. Actinomarina comprises ultra‐small, photoheterotrophic lineages restricted to the photic zone. Their streamlined genomes, together with proteorhodopsin‐like rhodopsins, also suggest high‐affinity uptake and turnover of low‐molecular‐weight DOM in low‐nutrient environments (Ghai et al. 2013; López‐Pérez et al. 2020). Marine Group II (MGII) archaea are also widely distributed in surface and DCM waters across the global ocean and can potentially degrade large molecules such as lipids, proteins and polysaccharides. Several MGII lineages harbour proteorhodopsins, and their abundances often increase during or after phytoplankton blooms, suggesting an important contribution to the post‐bloom remineralization of algal organic matter (Tully 2019; Pereira et al. 2019).

The NS5 marine group (Flavobacteria), on the contrary, are generally retrieved in large abundances when using 0.2‐μm filters and do not typically have particularly small cells (Priest et al. 2022; Haller et al. 2000; Elsaied et al. 2001; Obayashi and Suzuki 2019), which agrees with the detection of non‐negligible proportions of large cells (i.e., HNA) also in the FP fraction. Members of NS5 are commonly associated with phytoplankton blooms and have genomic and physiological traits enabling the use of phytoplankton‐derived organic matter (Priest et al. 2022; Teeling et al. 2016). Their presence in the FP fraction may thus indicate either their potential to miniaturize under non‐favourable conditions, or to their ability to pass through the 0.22 μm‐filter. For example, Elsaied et al. (2001) isolated a strain of the Bacteroidota genus *Microscilla* from the 0.2‐μm‐filtrate of seawater from Hiroshima Bay, which showed formation of small coccoid cells from larger filaments in late stationary phases. Conversely, other Bacteroidota genera such as *Flexibacter* and *Cytophaga* have been shown to exhibit 0.2 μm‐ and even 0.1 μm‐passable slender filamentous forms in seawater (Little et al. 1987) and freshwater (Hahn 2004), so it is also possible that some of the groups that we observed in the 0.1‐μm filters belong to this category of slender filamentous bacteria (*sensu* Nakai 2020).

Firmicutes and Parvibaculales were the only major groups identified as 0.1‐enriched, although they represented a small fraction of FP communities. Firmicutes appeared mostly in the FP fraction from Blanes Bay (Med_BBMO), where it comprised mostly the genus *Bacillus*. Firmicutes are spore‐forming bacteria that have been associated to airborne transport in the Mediterranean Sea (e.g., Gat et al. 2017) and the open ocean (Lang‐Yona et al. 2022), so it is possible that their presence was due to an event of atmospheric deposition and that they are not necessarily playing a role in the ecosystem. In groundwater, however, their enrichment in 0.2‐μm filtrates was attributed to their spiral shape with < 0.2 μm diameters that might allow them to pass through the filters (Herrmann et al. 2019). Regarding the Alphaproteobacteria Parvibaculales, very little is known about their ecology or cell size, yet the PS1 clade in particular has been reported within the microbiome associated to deep‐sea plastics (Kelly et al. 2022).

The fact that the vast majority of SVs were present in both size fractions and showed no clear size‐fraction preference further might indicate that a fraction of the FP pool may represent starvation forms of typical marine bacteria, which typically have small cell sizes. Cell size reduction is assumed to be a general phenomenon when prokaryotes are exposed to low nutrient or starvation conditions, and to occur frequently in aquatic environments (Velimirov 2001). For example, different heterotrophic bacterial isolates showed volumes decreasing up to 8‐fold after several days of starvation compared with optimal growth conditions (Lever et al. 2015; Herrmann et al. 2019). Although testing whether a fraction of our FP communities represent starvation forms would require experimental incubations, previous studies showed evidence of high growth potential and/or high amino acid uptake of the cells present in 0.2‐seawater filtrates from coastal Japan, the Caribbean and the Sargasso Sea (Li and Dickie 1985; Obayashi and Suzuki 2019), meaning that cells passing the filter retain the capacity to reactivate upon improved conditions. Such cells could potentially represent part of the so‐called ‘seed bank’ (Lennon and Jones 2011; Caporaso et al. 2012; Gibbons et al. 2013; Wisnoski and Lennon 2021). If dormant cells are indeed smaller than 0.2 μm, then previous studies on dormancy or rarity may have overlooked a fraction of these potential microbial seeds.

Remarkably, we found very low abundances of the ultra‐small Patescibacteria within our metabarcoding results (detected in only two 0.2RP and one FP samples at abundances below 0.03%), despite the fact that this group was identified by metagenomic sequencing of the FP fraction from the global Tara Oceans expedition (Lannes et al. 2019, 2021). It should be noted, though, that the latter sampling involved filtering much larger volumes. It is also known that most 16S rRNA primers are biased against Patescibacteria (Brown et al. 2015), including the ones used in our study (see Ruiz‐González et al. 2021). However, using this same primer pair we have detected Patescibacteria in nearby coastal seawater, several coastal groundwater sites and a hypersaline lagoon (e.g., Romano‐Gude et al. 2025). This suggests that, at least in the studied sites, Patescibacteria were either absent or very rare.

Finally, an indirect assessment of the functional potential using Tax4Fun2 indicated that, of the 16 KOs detected in our dataset (out of the 20 biogeochemically relevant genes highlighted by Auladell et al. 2023), only two KOs (*tau*A and *dmd*A) were enriched in the < 0.2 μm fraction. *tau*A, encoding the taurine ABC‐transporter substrate‐binding protein, is consistent with evidence that taurine is a major carbon and energy source for marine bacteria and is actively taken up by Pelagibacterales/SAR11 in epipelagic waters (Clifford et al. 2019). *dmd*A, on the other hand, encodes the DMSP demethylase that converts DMSP to methyl‐mercaptopropionate (MMPA), enabling the assimilation of reduced sulfur and carbon through a pathway that is widespread and predominantly hosted by Pelagibacterales in surface waters (Reisch et al. 2008; Nowinski et al. 2019). Hence, this association with Pelagibacterales likely explains their preferential presence in FP communities, enriched in this group. In any case, a more definitive assessment of the functional potential of filterable prokaryotes would require a direct exploration of all genes encoded in this size fraction using metagenomics or metatranscriptomics, given that KO profiles inferred from 16S rRNA data are indirect and constrained by reference‐genome coverage and phylogenetic assumptions.

### 4.3 Organic Carbon Availability May Shape FP Community Structure

Overall, we did not find evidence of a differential structuring of 0.2RP versus FP assemblages at the community level. In both cases, the relationships between taxonomic dissimilarity and environmental differences across samples were similar, with salinity and temperature showing the strongest correlations to community shifts. Although increases in temperature may lead to an overall size reduction in marine prokaryotes (Morán et al. 2015; Huete‐Stauffer et al. 2016), we did not find evidence of a clear effect of temperature on FP abundance or cell size. However, we did find that the relative proportion of the ultra‐small Actinobacteria (mostly Cand. *Actinomarina*) increased towards warmer waters in both size‐fractions, and proportionally more in FP samples. Conversely, the proportion of Flavobacteriales (normal‐sized groups, mostly NS5 marine group) decreased equally with increasing temperature in both size fractions.

In other cases, however, the relationship with environmental variables was not always the same in both size‐fractions, as exemplified by Pelagibacterales, which showed opposite behaviours with TOC in each size‐fraction. It is thus likely that, in these cases, the FP sub‐pool is not an accurate representation of the 0.2RP community, and hence that by using the 0.22 μm filter we are missing a potentially ecologically relevant fraction of the diversity within these groups. Further studies covering larger spatial or temporal gradients will be required to determine to which extent FP communities hide ecological patterns that are missed with common sampling strategies.

In terms of the relative proportions of SVs enriched in each size fraction, we found that the relative abundance of 0.2‐enriched SVs on the 0.2 μm filters increased with higher TOC concentrations in the water. In contrast, FP communities showed a greater contribution of 0.1‐enriched SVs at sites with elevated nitrate levels. This resulted in a broader pattern in which the taxonomic dissimilarity between the two size fractions was greater at sites with the highest TOC concentrations (e.g., Atl_D2 and Med_BBMO) compared to those with lower TOC, such as the Red Sea samples collected in summer. Although the reason behind this pattern is unclear, the bacterial biovolume is often positively related to the availability of DOC (e.g., Church et al. 2000), and organic carbon depletion is known to translate into large reductions in cell size of heterotrophic bacteria (Herrmann et al. 2019). It is thus possible that in situations of DOC limitation, a larger fraction of typically normal‐sized prokaryotes is miniaturized and hence FP are more similar to the > 0.22‐assemblages. Conversely, if DOC is less limiting, most potentially miniaturizable taxa would be large, and FP communities would be enriched in truly ultra‐small prokaryotic groups, being more distinguishable from the large size fraction. To our knowledge, this is the first study comparing prokaryotic communities retrieved onto 0.2 and 0.1 μm filters across environmentally different coastal sites. Although we acknowledge that our sampling encompassed only four locations, and hence the patterns observed may not be broadly extrapolable to other marine environments, the variations observed point to a relatively dynamic pool of overlooked ‘filterable’ prokaryotes that may harbour relevant ecological roles in marine systems.

## 5 Conclusions

Our results show that microbial biomass collection in 0.22‐μm pore size filters may disregard a significant fraction of marine prokaryotic communities. This pool of filterable taxa was found to be enriched in small‐sized groups such as Pelagibacterales, Candidatus *Actinomarina* and the archaeal Marine Group II across the four studied coastal sites. A significant proportion of the bacteria found in this fraction might also be starvation forms of groups with larger cell sizes, such as Bacteroidota (mostly NS5 marine group) and Gamma‐ and Alphaproteobacteria, but this remains to be verified. The most abundant filterable SVs were also retrieved in the 0.22‐μm filters, meaning that, in terms of richness, common filtration strategies may not be disregarding a large fraction of the diversity. However, globally dominant groups such as Pelagibacterales were enriched in 0.1 μm‐communities, and the SVs retrieved in each size fraction showed different relationships with environmental factors, suggesting that we may be losing certain ecological information of small‐sized groups. Finally, the degree of taxonomic differentiation between 0.22‐ and 0.1 μm‐assemblages appeared related to TOC concentration, perhaps due to a different degree of taxa miniaturization following OC limitation. Spatial or temporal environmental variations, such as changes in temperature or OC concentration, may affect the number or the identity of cells that escape 0.2‐μm filtration and thus, incorporating this filterable cell pool in microbial ecology studies may become increasingly relevant.

## Author Contributions

X.A.G.M. conceived the main idea of the manuscript. All authors participated in the design of the samplings and collected samples in the different sites. C.A.‐B. optimized the sampling protocol and processed all the samples. A.L. performed all bioinformatic processing of the raw sequences, and C.R.‐G. conducted all data analyses, prepared all figures and wrote the manuscript with valuable contributions of all coauthors.

## Funding

This work acknowledges the funding of projects MARUMBA (KAUST Competitive Research Grant program), MINIOM (PID2022‐142480NB‐I00) and MicroSub (SPIP2020‐02595) funded by the Spanish Ministry of Science Innovation and Universities (MICINN). A.L. is supported by a Research Assistant Professorship from IKERBASQUE (Basque Foundation for Science). The institutional support of the ‘Severo Ochoa Centre of Excellence’ accreditation (CEX2024‐001494‐S) is also acknowledged. C.R.‐G. and L.A.‐S. were supported by the Ramon y Cajal contracts RYC2019‐026758‐I and RYC‐2012‐11404 funded by MICINN, and C.A.‐B. by the KAUST CRG program.

## Ethics Statement

The authors have nothing to report.

## Conflicts of Interest

The authors declare no conflicts of interest.

## Supporting information


**Table S1:** Number of replicate filters for DNA analyses collected for each of the size‐fractions during the different samplings, and volume filtered per replicate.
**Table S2:** Relative abundance (% of community reads) of SVs exclusively found on 0.2 μm pore‐sized filters (0.2RP‐unique) or on 0.1 μm pore‐sized filters (FP‐unique). Values are averages ± standard deviation of the replicates.
**Table S3:** R coefficients of the Mantel correlations performed between differences in environmental parameters and Bray‐Curtis dissimilarities between 0.2RP or FP communities. The strongest significant correlations (i.e., those with *p* < 0.001) are highlighted in bold.
**Figure S1:** Location of the sampling sites in the Bay of Biscay (Atlantic, Atl_G2 and Atl_D2), the Blanes Bay Microbial Observatory in the Mediterranean Sea (Med_BBMO) and the Red Sea (corresponding to samplings Red_Sep, Red_Oct, Red_Feb, given that three samplings were conducted on different occasions in this site, see Table 1 in main manuscript).
**Figure S2:** Taxonomic composition of the groups that dominated FP communities (Actinobacteriota, Thermoplasmatota, Flavobacteriales and Pelagibacteriales), including Firmicutes, which was abundant in FP from Med_BBMO and Parviculales, which were abundant among the 0.1‐enriched taxa identified in the Red Sea samples. nd, not detected.
**Figure S3:** Relative abundance of the 16 biogeochemically relevant KOs selected following Auladell et al. (2023) between the two size fractions, based on the full set of KOs predicted with Tax4Fun2 from our ASVs (see Methods). Detailed descriptions of each KO are provided in Figure 5.
**Figure S4:** Heatmap of correlations between the measured physicochemical conditions and the abundance and average cell size of total prokaryotes, of those passing the 0.22‐μm filter (FP), proportion of FP cells (% FP), difference in average cell size between total prokaryotes and FP cells (Diff in cell size), as well as the taxonomic richness of both size fractions (0.2RP and FP richness). The colour gradient indicates the Spearman correlation coefficients (Rho values). The asterisks (*) indicate significant relationships (*p* < 0.05, *n* = 6). TOC, total organic carbon concentration; Chl, chlorophyll *a* concentration; TN, total dissolved nitrogen; sal, salinity; temp, temperature; silica, dissolved silicate concentration.
**Figure S5:** Examples of filter membranes with pores exceeding their nominal size. Scanning electron micrographs of marine cells from Blanes Bay retained on 0.2 μm (polycarbonate, GTTP, Merck Millipore, left pannel) and 0.1 μm (VCTP, Merck Millipore, right pannel) pore‐size filters. Yellow arrows highlight pores visibly larger than the nominal size, which could potentially allow large cells to pass through. The 0.2 μm filter was likely defective, as pores are generally not expected to deviate so markedly from their nominal size and we have only occasionally observed such irregular pores. The sampling time and filters used differ from those applied in the present study.

**Table S4:** SV table.

**Table S5:** Taxonomic classification of SVs.

**Table S6:** Environmental metadata. Note that the meaning of each variable is provided in the second sheet.

**Table S7:** KO abundances per sample.

**Table S8:** Functional annotation table derived from Tax4Fun2, including KEGG Orthologs (KO), gene descriptions, relative abundances (M0), associated genes, pathways and hierarchical metabolic classifications (Metabolism levels 1 and 2).

## Data Availability

Raw sequence data are publicly available in the Sequence Read Archive (www.ncbi.nlm.nih.gov/sra) under accession number (PRJEB86314). The complete non‐rarefied SV table, taxonomy table and environmental data used in this study are provided as Tables S4–, S6, and the tables containing KO abundance and descriptions are provided as Tables S7 and S8.
