# Supplementary material for: The Elusive Fraction of Marine Plankton Diversity: Size, Abundance and Taxonomic Composition of 0.2 μm‐Filterable Prokaryotes Across Three Contrasting Coastal Environments
Source: Mol Ecol. 2026 Jun 15;35(12):e70434. doi: 10.1111/mec.70434 (PMC13266375; doi:10.1111/mec.70434)
Supplement: Supplementary file 1 — Table S1: Number of replicate filters for DNA analyses collected for each of the size‐fractions during the different samplings, and volume filtered per replicate. Table S2: Relative abundance (% of community reads) of SVs exclusively found on 0.2 μm pore‐sized filters (0.2RP‐unique) or on 0.1 μm pore‐sized filters (FP‐unique). Values are averages ± standard deviation of the replicates. Table S3: R coefficients of the Mantel correlations performed between differences in environmental parameters and Bray‐Curtis dissimilarities between 0.2RP or FP communities. The strongest significant correlations (i.e., those with p < 0.001) are highlighted in bold. Figure S1: Location of the sampling sites in the Bay of Biscay (Atlantic, Atl_G2 and Atl_D2), the Blanes Bay Microbial Observatory in the Mediterranean Sea (Med_BBMO) and the Red Sea (corresponding to samplings Red_Sep, Red_Oct, Red_Feb, given that three samplings were conducted on different occasions in this site, see Table 1 in main manuscript). Figure S2: Taxonomic composition of the groups that dominated FP communities (Actinobacteriota, Thermoplasmatota, Flavobacteriales and Pelagibacteriales), including Firmicutes, which was abundant in FP from Med_BBMO and Parviculales, which were abundant among the 0.1‐enriched taxa identified in the Red Sea samples. nd, not detected. Figure S3: Relative abundance of the 16 biogeochemically relevant KOs selected following Auladell et al. (2023) between the two size fractions, based on the full set of KOs predicted with Tax4Fun2 from our ASVs (see Methods). Detailed descriptions of each KO are provided in Figure 5. Figure S4: Heatmap of correlations between the measured physicochemical conditions and the abundance and average cell size of total prokaryotes, of those passing the 0.22‐μm filter (FP), proportion of FP cells (% FP), difference in average cell size between total prokaryotes and FP cells (Diff in cell size), as well as the taxonomic richness of both size fractions (0.2RP [file MEC-35-e70434-s004.docx]

**Supplemental Information for:**

**The elusive fraction of marine plankton diversity: size, abundance and taxonomic composition of 0.2 µm-filterable prokaryotes across three contrasting coastal environments**

Clara Ruiz-González^*^, Cristina Andres-Barrao^*^, Anders Lanzén, Laura Alonso-Sáez, Hugh Ducklow, Josep M. Gasol, Xosé Anxelu G. Morán

**Table of Contents:**

| **Supplementary Tables S1 and S2** | Page 2 |
| --- | --- |
| **Supplementary Table S3** | Page 3 |
| **Supplementary Figure 1** | Page 4 |
| **Supplementary Figure 2** | Page 5 |
| **Supplementary Figure 3** | Page 6 |
| **Supplementary Figure 4** | Page 7 |
| **Supplementary Figure 5** | Page 8 |

**Supplementary Table S1.** Number of replicate filters for DNA analyses collected for each of the size-fractions during the different samplings, and volume filtered per replicate.

| Site ID | # of replicates | Filtered volume (L) |
| --- | --- | --- |
| Med_BBMO | 2 | 1.5 |
| Atl_D2 | 3 | 1 |
| Atl_G2 | 3 | 10 |
| Red_Sep | 2 | 5 |
| Red_Oct | 4 | 5 |
| Red_Feb | 3 | 5 |

**Supplementary Table S2.** Relative abundance (% of community reads) of SVs exclusively found on 0.2 µm pore-sized filters (0.2RP-unique) or on 0.1µm pore-sized filters (FP-unique). Values are averages ± standard deviation of the replicates.

|  | **%0.2RP-unique reads** | **% FP-unique reads** |
| --- | --- | --- |
| Atl_D2 | 2.5 ± 1.4 | 0.1 ± 0.8 |
| Atl_G2 | 2.3 ± 0.2 | 0.1 ± 0.1 |
| Med_BBMO | 5.3 ± 0.2 | 1.4 ± 0.9 |
| Red_Oct | 1.4 ± 0.2 | 0.2 ± 0.1 |
| Red_Sep | 1.8 ± 0.6 | 0.1 ± 0.1 |
| Red_Feb | 4.5 ± 0.6 | 0.4 ± 0.2 |

**Suppl. Table S3 |** R coefficients of the Mantel correlations performed between differences in environmental parameters and Bray-Curtis dissimilarities between 0.2RP or FP communities. The strongest significant correlations (i.e. those with p<0.001) are highlighted in **bold.**

|  | Mantel R | |
| --- | --- | --- |
|  | 0.2RP -communities | FP-communities |
| Temperature | **0.709** | **0.651** |
| Salinity | **0.827** | **0.803** |
| TOC | **0.535** | **0.496** |
| Total nitrogen | **0.406** | 0.356 |
| Nitrate | 0.296 | **0.409** |
| Nitrite | 0.171 | 0.312 |
| Phosphate | 0.368 | 0.303 |
| Silicate | 0.243 | 0.222 |
| Chlorophyll | 0.331 | 0.325 |

**
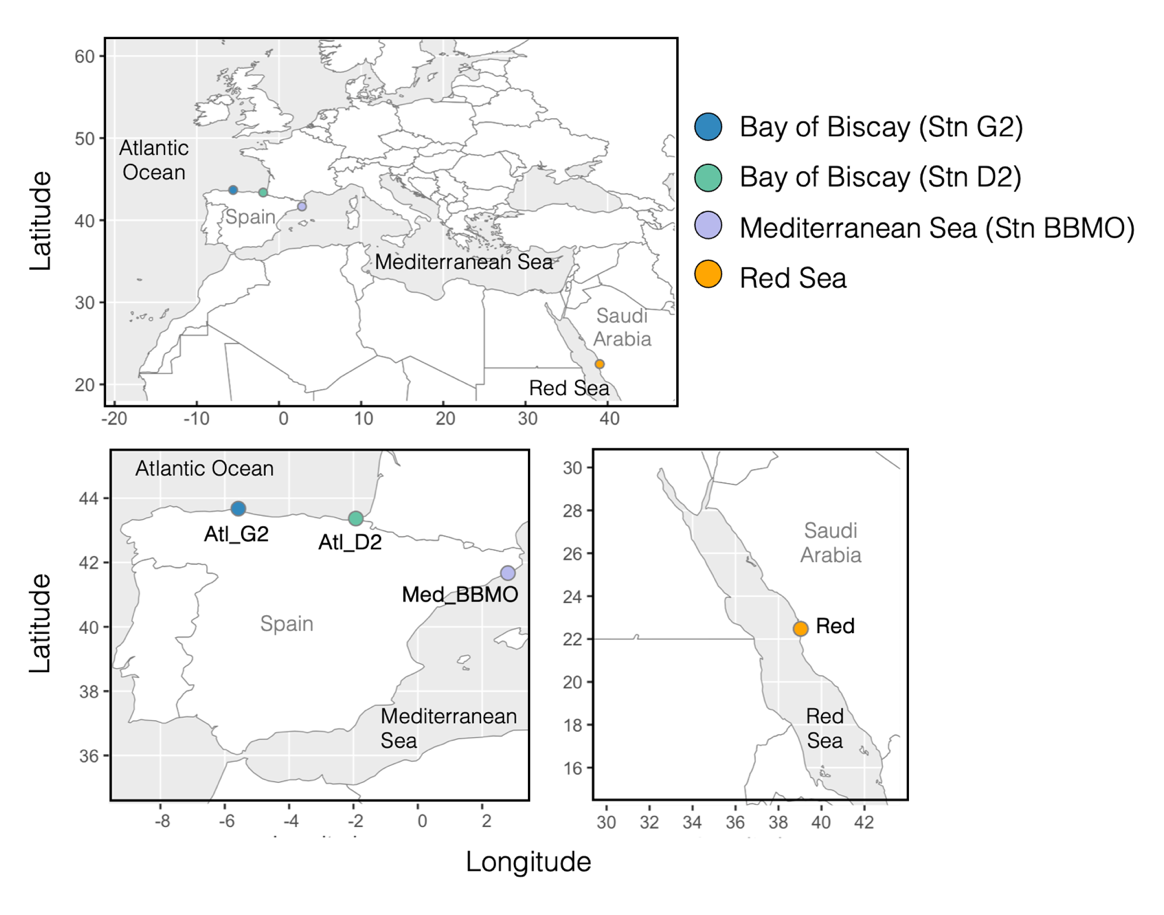
**

**Supplementary Figure 1**. Location of the sampling sites in the Bay of Biscay (Atlantic, Atl_G2 and Atl_D2), the Blanes Bay Microbial Observatory in the Mediterranean Sea (Med_BBMO) and the Red Sea (corresponding to samplings Red_Sep, Red_Oct, Red_Feb, given that three samplings were conducted on different occasions in this site, see Table 1 in main manuscript).


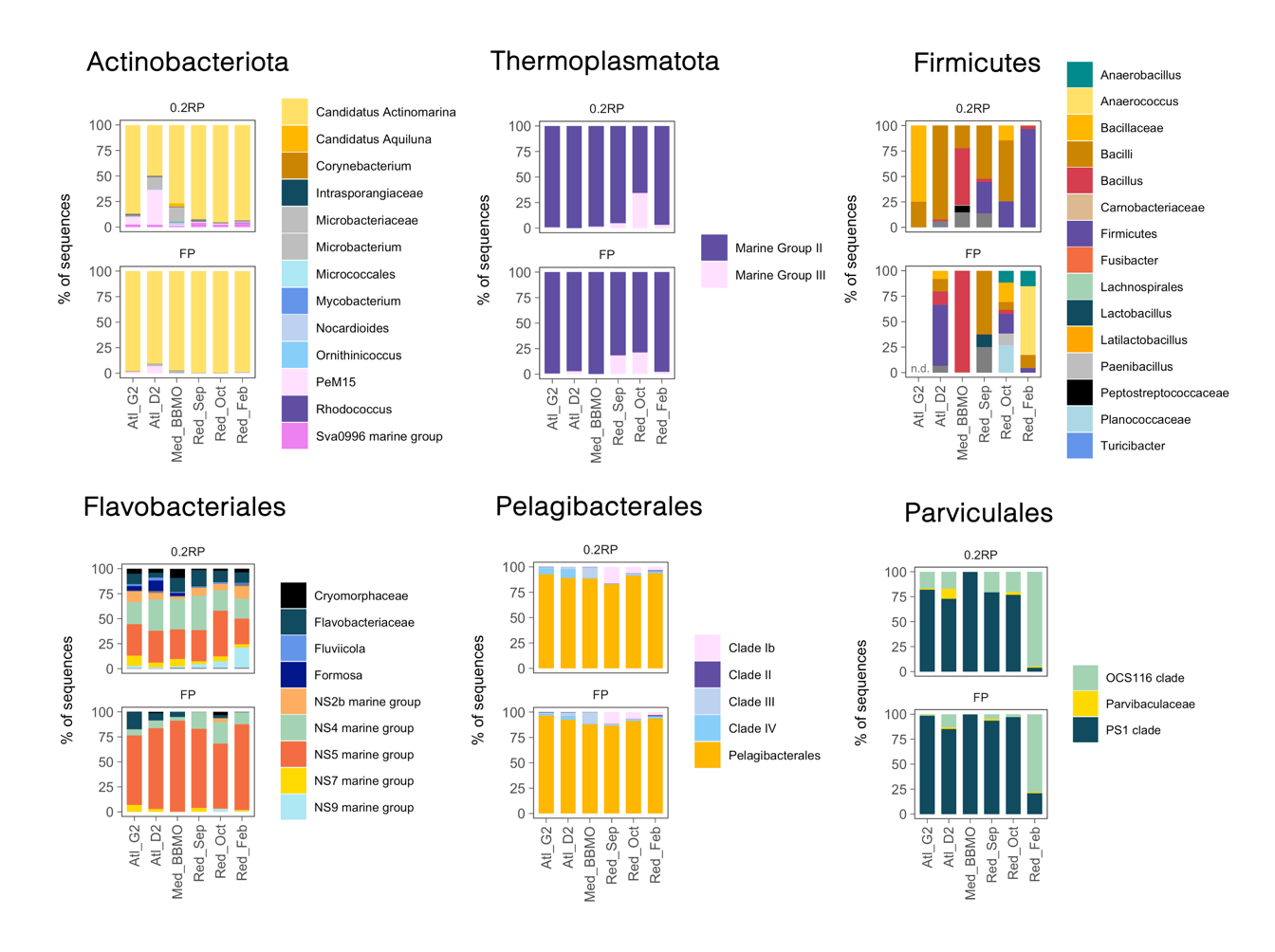


**Supplementary Figure 2.** Taxonomic composition of the groups that dominated FP communities (Actinobacteriota, Thermoplasmatota, Flavobacteriales and Pelagibacteriales), including Firmicutes, which was abundant in FP from Med_BBMO, and Parviculales, which were abundant among the 0.1-enriched taxa identified in the Red Sea samples. n.d, not detected.


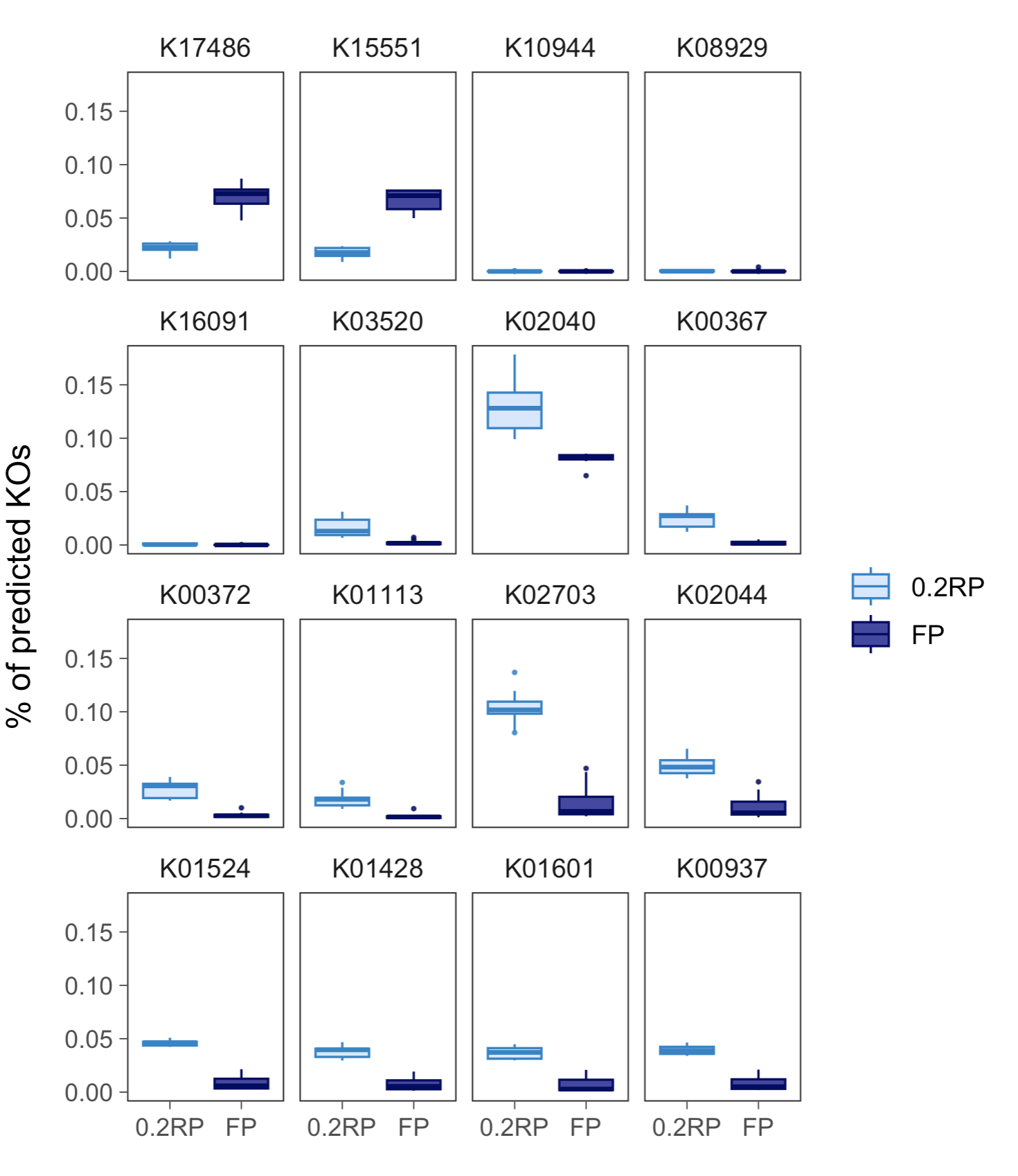


**Supplementary Figure 3.** Relative abundance of the 16 biogeochemically relevant KOs selected following Auladell *et al.* (2023) between the two size fractions, based on the full set of KOs predicted with Tax4Fun2 from our ASVs (see Methods). Detailed descriptions of each KO are provided in Figure 5.


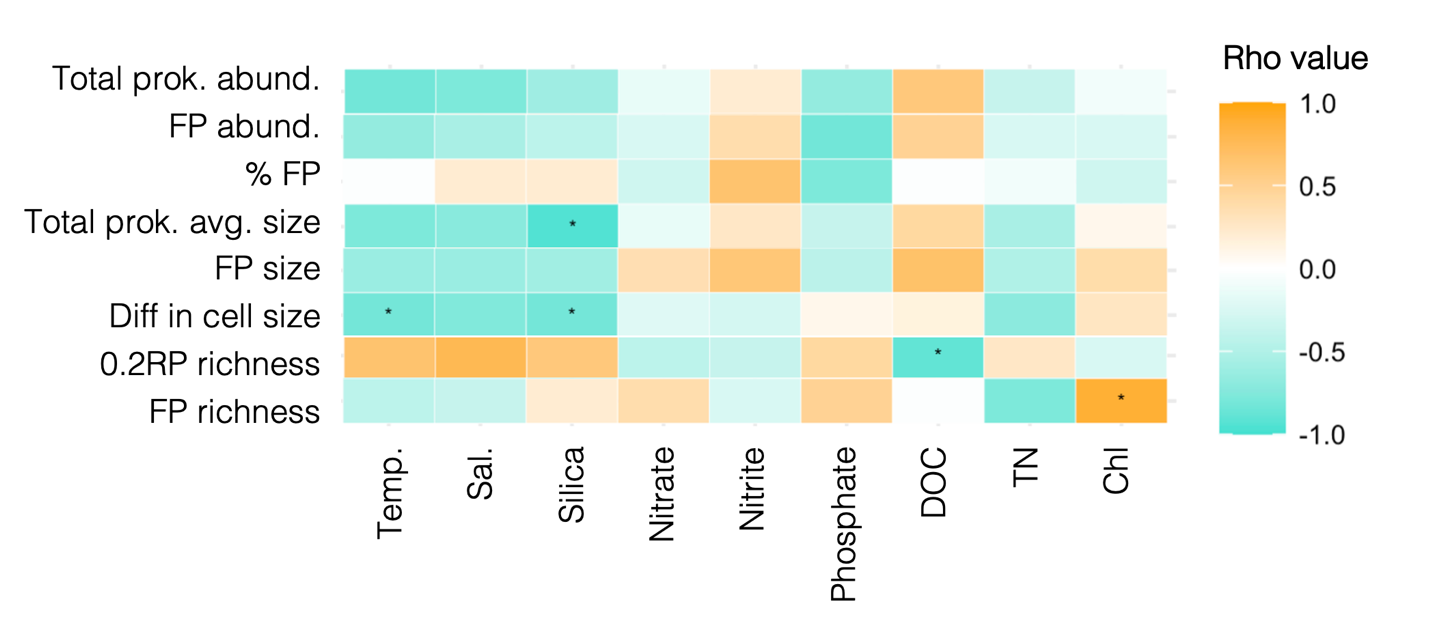


**Supplementary Figure 4.** Heatmap of correlations between the measured physicochemical conditions and the abundance and average cell size of total prokaryotes, of those passing the 0.22-μm filter (FP), proportion of FP cells (% FP), difference in average cell size between total prokaryotes and FP cells (Diff in cell size), as well as the taxonomic richness of both size fractions (0.2RP and FP richness). The colour gradient indicates the Spearman correlation coefficients (Rho values). The asterisks (*) indicate significant relationships (p < 0.05, n=6). TOC, total organic carbon concentration; Chl, chlorophyll *a* concentration; TN, total dissolved nitrogen; sal, salinity; temp, temperature; silica, dissolved silicate concentration.


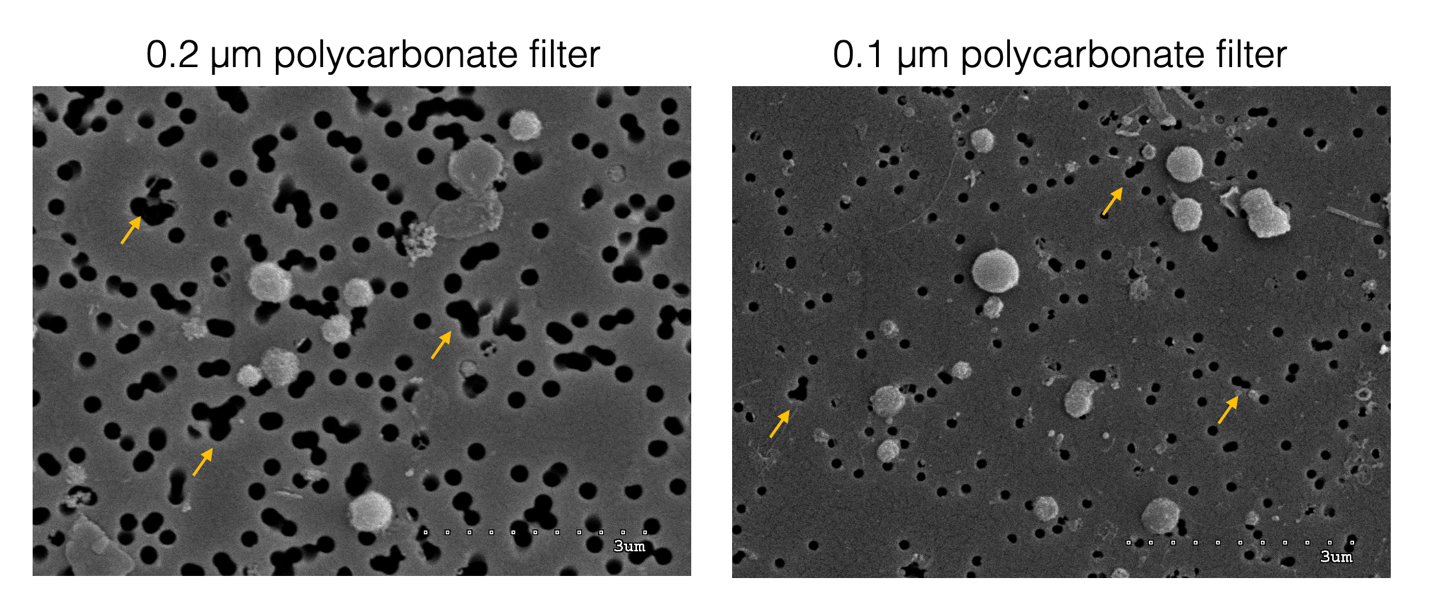


**Supplementary Figure 5.**  **Examples of filter membranes with pores exceeding their nominal size.** Scanning electron micrographs of marine cells from Blanes Bay retained on 0.2 µm (polycarbonate, GTTP, Merck Millipore, left pannel) and 0.1 µm (VCTP, Merck Millipore, right pannel) pore-size filters. Yellow arrows highlight pores visibly larger than the nominal size, which could potentially allow large cells to pass through. The 0.2 µm filter was likely defective, as pores are generally not expected to deviate so markedly from their nominal size and we have only occasionally observed such irregular pores. The sampling time and filters used differ from those applied in the present study.
